# Degradation of Glyphosate to Benign *N*‐Formyl Glycine Using MOF‐808 Nanocrystals

**DOI:** 10.1002/anie.202424540

**Published:** 2025-03-17

**Authors:** Jhair A. Peña Prada, Tatiana A. Huertas Navarro, Stephanie L. Chua, Alejandro M. Granados, Chih‐Wen Pao, Alejandro M. Fracaroli, Nicholas M. Bedford

**Affiliations:** ^1^ School of Chemical Engineering The University of New South Wales Sydney New South Wales 2052 Australia; ^2^ Departamento de Química Orgánica Facultad de Ciencias Químicas, Universidad Nacional de Córdoba (UNC) Inst. de Inv. en Fisicoquímica de Córdoba (INFIQC‐CONICET) X5000HUA Córdoba Argentina; ^3^ National Synchrotron Radiation Research Center Hsinchu 30076 Taiwan; ^4^ Department of Chemistry Colorado School of Mines Golden CO 80401 USA

**Keywords:** Glyphosate degradation, Heterogeneous catalysis, Metal–organic frameworks, nMOF‐808

## Abstract

Glyphosate (*N*‐phosphonomethyl glycine, GPh) is an industrial herbicide used worldwide in modern agricultural practices. With the growing concerns regarding cumulative environmental and health effects, pathways for catalytic GPh degradation to benign products are becoming a pressing societal need. This report demonstrates that Zr‐based metal–organic framework (MOF‐808) with different crystal sizes and designed defect sites can be employed as an efficient heterogeneous catalyst for the complete degradation of GPh at room temperature. Importantly, the degradation mechanism produces *N*‐formyl glycine and hydroxymethyl‐phosphonate, which are largely innocuous chemicals, especially when compared to more common GPh degradation products. Nanocrystalline MOF‐808 (nMOF‐808) exhibits enhanced reactivity than larger MOF‐808 crystals, attributed to the higher coordination of hydroxyl and water molecules to the secondary building units (SBU) as determined using a range of X‐ray absorption spectroscopy (XAS) techniques. These studies indicate that the crystal size‐dependency in GPh degradation is related to structural modifications on coordinative unsaturated Zr site that promote the fast exchange of weakly bonded ligands. Taken together, this study demonstrates that GPh degradation can be optimized through ligand field tuning in MOFs, which can help improve overall reactivity while also pushing the reaction toward desirable, nontoxic products.

## Graphical abstract

A Zr‐based metal–organic framework (MOF‐808) with different crystal sizes and designed defect sites was employed as an efficient heterogeneous catalyst for the complete degradation of glyphosate at room temperature. The degradation mechanism produces *N*‐formyl glycine and hydroxymethyl‐phosphonate.

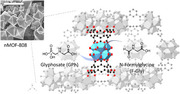

## Introduction

**Figure 1:**
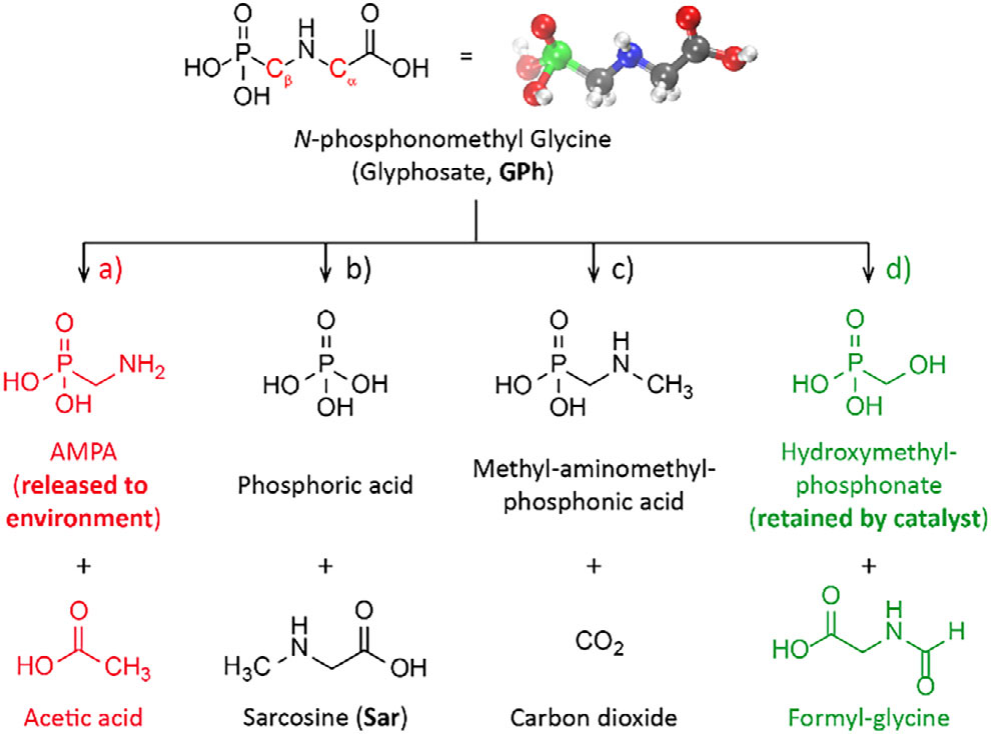
Chemical structure of Gph. Potential degradation pathways: a) microbial degradation to AMPA, b) sarcosine formation, c) CO_2_ release, and d) *N*‐formyl glycine.

Glyphosate (GPh, Figure 1) and its derivatives are the most widely used nonselective herbicides worldwide.^[^ ^1^ ^]^ Due to its extensive use, there are concerns about GPh bioaccumulation,^[^ ^2^ ^]^ toxicity, and their persistence in food products.^[^ ^3^ ^]^ For example, studies suggest a carcinogenic potential of GPh associated to the contribution effects of all components in various commercial formulations.^[^ ^4^ ^]^ Moreover, the broadband efficacy of GPh is known to affect natural vegetation as well as some fungi and bacteria in the environment.^[^ ^5^ ^]^ Considering these factors, different remediation processes have been studied to remove GPh and its derivatives from the environment, including adsorption technologies and degradation modalities.^[^ ^6^ ^]^

Although various adsorption and capture methodologies exhibit promising results,^[^ ^7^, ^8^ ^]^ most notably resin D31 (833.33 mg.g^−1^),^[^ ^9^ ^]^ issues with the generation of secondary wastes, and cost at scale are persistent issues.

Degradation pathways provide another mode GPh remediation that could, in principle, lead to the removal of the pesticide with minimal secondary waste if environmentally innocuous products are a result of the process. Unfortunately, several observed GPh degradation products are themselves harmful to the environment,^[^ ^10^, ^11^ ^]^ lessening the viability of degradation‐based remediation strategies. For example, popular microbial bioremediation strategies^[^ ^12^ ^–‐^ ^15^ ^]^ and hydrolysis over metal oxides^[^ ^16^ ^–‐^ ^19^ ^]^ show that GPh follows two general mechanisms: 1) the selective breaking of the C_α_─N bond to produce amino methyl phosphonic acid (AMPA) or 2) the breaking of the C_β_─P bond to generate sarcosine and phosphoric acid (Figure 1a,b). These are nonideal degradation pathways as AMPA has demonstrated toxicity on human cells,^[^ ^20^ ^]^ whereas phosphoric acid is known to contribute to the eutrophication of freshwater.^[^ ^21^ ^]^ Computational chemistry efforts have demonstrated that GPh degradation can occur at C─C bonds (Figure 1c), although this has not been verified experimentally.^[^ ^22^ ^]^ More emerging methods for GPh degradation, including (electro)oxidation and photodriven processes,^[^ ^23^ ^–‐^ ^30^ ^]^ are broadly nonselective, wherein reactive oxygen species (ROS) are used to generate a range of compounds. As such, it is paramount that GPh remediation processes that result in captured phosphorous products and nontoxic byproduct moieties be emphasized to truly achieve environmental decontamination. In this context, high absorbent and catalytic materials like metal–organic frameworks (MOFs) are advantageously positioned for such applications. Previous work describe the capture of GPh for a range of MOFs after 48 h of exposure,^[^ ^31^ ^–‐^ ^36^ ^]^ most notably metacarborane‐modified MOF‐2 (≤1.9 g.g^−1^ (GPh/MOF)),^[^ ^37^ ^]^ whereas photocatalytic MOFs were found to be highly selective toward glycine^[^ ^38^ ^]^ and sarcosine.^[^ ^37^ ^]^ Direct GPh degradation with MOFs, however, is relatively unexplored.

In this effort, Zr‐based MOFs were engineered with variation to the local Zr active site to enhance GPh degradation reactions. Inspired by the activity of GPh degradation in phosphatases,^[^ ^39^, ^40^ ^]^ MOF‐808 was synthesized at two different crystal sizes, MOF‐808 (≈600 nm) and nMOF‐808 (≈65 nm), based on the ability of smaller MOF crystals to promote adsorption and exhibit a higher degree of defective topologies that can act as active sites.^[^ ^36^, ^41^ ^]^ Our results showed a degradation capacity of 95% after 2 h using a 10%  mol ratio of nMOF‐808, equivalent to an adsorption capacity of GPh/nMOF‐808, 1.1 g.g^−1^. Such unusual adsorbed amounts in a short period indicate that not only adsorption but a reaction is occurring, which is confirmed by the evidence of products. Additionally, GPh degradation was evaluated in two consecutive cycles without significant changes in their effectiveness, indicating its potential for reusability. Degradation properties and efficiency were thoroughly examined using nuclear magnetic resonance (NMR), high performance liquid chromatography–mass spectrometry (HPLC–MS), X‐ray absorption spectroscopy (XAS), total scattering, and Fourier transform infrared spectroscopy (FT‐IR). Importantly, the observed decomposition pathway of GPh yields low‐toxicity products: hydroxymethyl‐phosphonate (retained by the catalyst) and *N*‐formyl glycine (F‐Gly, Figure 1d), without requiring any external stimuli. These findings demonstrate a route toward complete GPh remediation, where its degradation products result in minimal harm to the environment while further showcasing the importance of catalysts engineering through synthetic modulation to achieve desired outcomes.

## Results and Discussion

Decomposition of organophosphate compounds (OPCs) by Zr‐MOFs is widely reported and is commonly agreed that the reaction is favored by the fast exchange of labile ligands such as molecular water and hydroxyl groups.^[^ ^42^ ^–‐^ ^44^ ^]^ The interaction of the Zr‐secondary building units (Zr‐SBU) with OPCs is enhanced by large pores that facilitate diffusion, jointly with unsaturated coordinative sites that account for an enhanced Lewis acidity.^[^ ^45^, ^46^ ^]^ Moreover, MOF activity can be modulated through restricted crystal growth strategies that promotes the presence of unsaturated coordinative sites^[^ ^47^, ^48^ ^]^ and higher diffusion rates through the MOF.^[^ ^36^ ^]^

**Figure 2:**
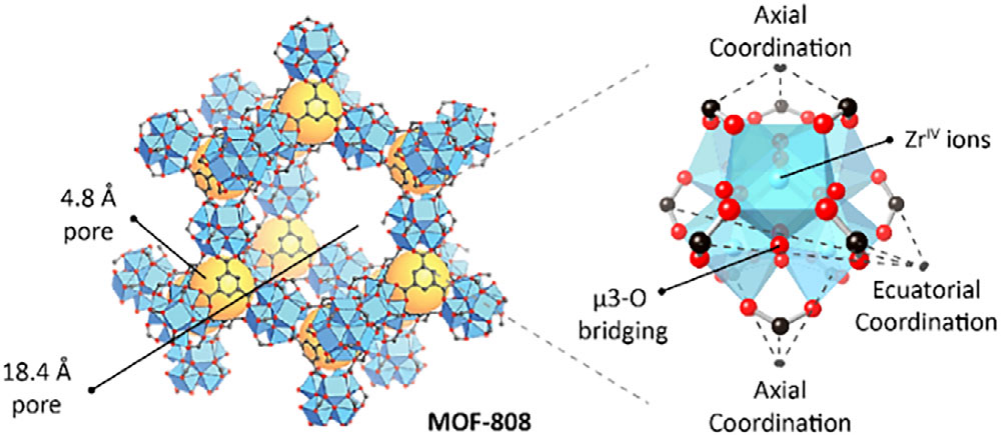
Schematic representation of MOF‐808 with its Zr‐SBU highlighting the axial and equatorial coordination sites.

MOF‐808 [Zr_6_O_4_(OH)_4_(BTC)_2_(HCOO)_6_, BTC = 1,3,5‐benzenetricarboxylate, F.W. = 1363.7 g.mol^−1^] (Figure 2)^[^ ^49^ ^]^ was identified as an ideal GPh absorbent and heterogenous catalytic material as it features a low coordinated Zr‐SBU connected by six tritopic organic linkers (benzene 1,3,5‐tricarboxylate; BTC) in the axial positions of the metal cluster, termed secondary building units (SBUs). The remaining coordinative sites are located at the equatorial region and populated with terminal ligands from the synthetic procedure (formic or acetic acid), and/or organic solvent molecules used during activation steps (please refer to MOF‐808 synthesis section, Supporting Information). One of the key aspects of MOF‐808 is its chemical stability in water, which can further withstand a wide variety of pH ranges.^[^ ^50^ ^]^ After MOF‐808 synthesis, the terminal ligands located in the equatorial positions of the Zr‐SBU can be removed efficiently, leaving behind coordinative unsaturated sites that can be used as anchoring points for different active molecules^[^ ^51^ ^]^ or simply as heterogeneous catalytic sites. Considering the importance of the coordinatively unsaturated sites for GPh adsorption/decomposition, extended defective sites in the MOF solids were probed by preparing MOF‐808 nanocrystals, denoted nMOF‐808, using a modified synthetic procedure^[^ ^52^ ^]^ (refer to nMOF‐808 synthesis section, Supporting Information).

**Figure 3:**
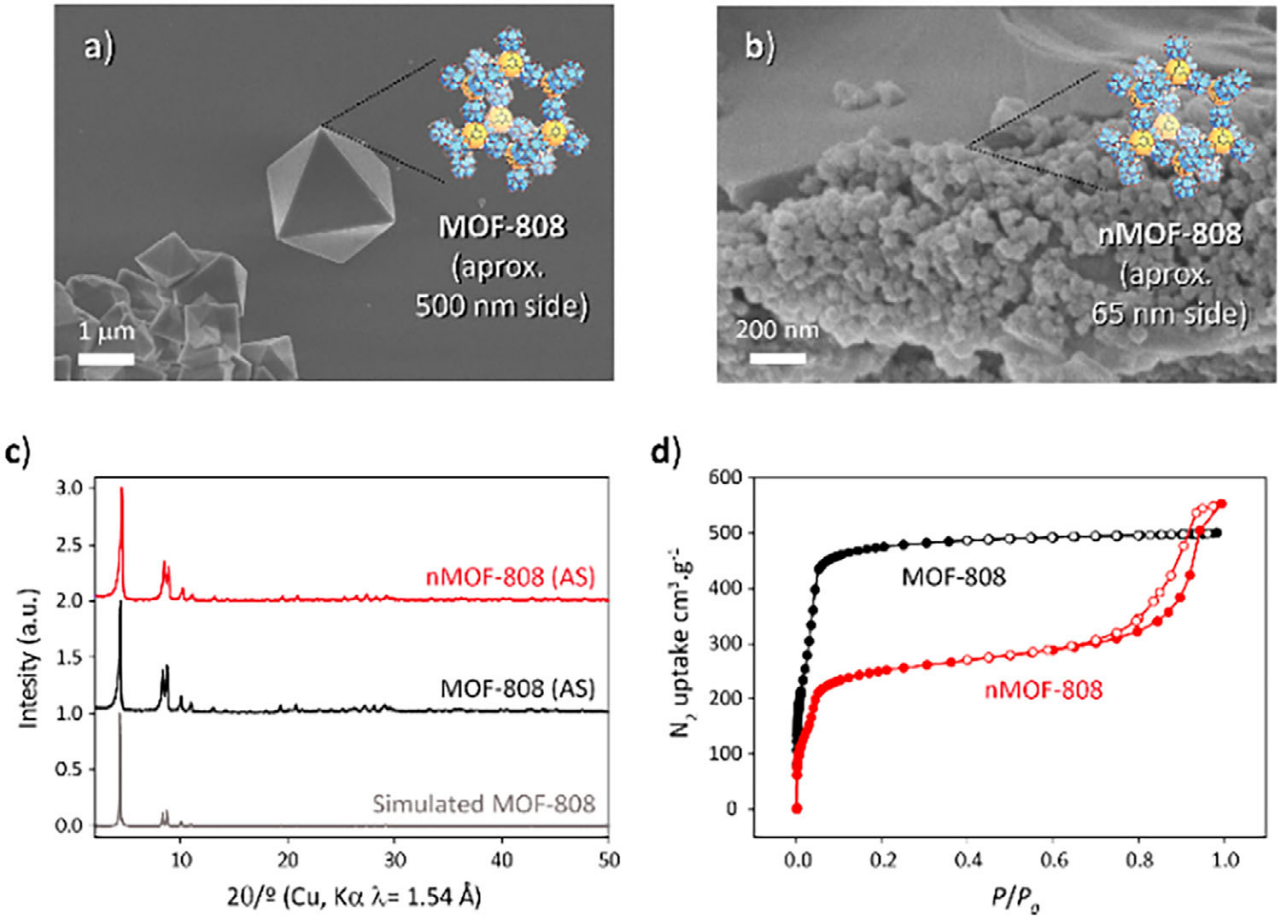
SEM images of a) MOF‐808 and b) nMOF‐808. c) PXRD patterns comparison with AS (AS, as synthetized), and d) N_2_ adsorption isotherms for MOF‐808 (black) and nMOF‐808 (red). Full circles in each isotherm correspond to adsorption curves, whereas empty circles correspond to desorption points.

Scanning electron microscopy (SEM) images (Figures 3a,b) display octahedral crystal shapes for both crystal sizes prepared, with MOF‐808 exhibiting a crystal size of ≈600 nm and nMOF‐808 showcasing a much smaller crystal size of ≈65 nm. The crystallinity and connectivity of the structure were initially evaluated by powder X‐ray diffraction (PXRD). The coincidence of the sharp diffraction lines with the simulated pattern from the literature^[^ ^49^ ^]^ allowed us to confirm the structures of MOF‐808 (Figure 3c), showcasing the long‐range periodicity of MOF‐808. Nitrogen adsorption isotherms were measured at 77 K, and the Brunauer−Emmett−Teller (BET) surface areas were calculated to be 1570 m^2^.g^−1^ and 848 m^2^.g^−1^ for MOF‐808 and nMOF‐808, respectively (Figure 3d). A hysteresis loop can be observed for nMOF‐808 isotherm at 0.8 relative pressure (Figure 3d), which can be attributed to defects in the structure attributed to the two‐step crystal growth synthetic procedure used (see nMOF‐808 synthesis section, Supporting Information).

The interaction of GPh with MOF‐808 was evaluated in D_2_O to be able to follow the reaction progress using NMR analyses. In a typical reaction, 3.9 mg of GPh were dissolved in D_2_O (1 mL) and added to a 20 mL scintillation vial jointly along with 10%  mol of MOF relative to GPh (3.5  mg of MOF). This mixture was kept at room temperature for 2 h under constant stirring. During this period, we were able to observe the course of the GPh degradation through the area‐reduction of its assigned signals (^1^H NMR in D_2_O: *δ* = 3.25 ppm, doublet; and δ = 3.96 ppm, singlet, Figure S1). These results allowed us to calculate a 95% and 72% degradation of GPh for nMOF‐808 and MOF‐808, respectively, under these conditions (TON of 8.55 and 6.48 of nMOF‐808 and MOF‐808 per catalytic cycle, TON = *n*_product_/*n*_catalyst_, see Figure S1). To validate this analysis, we also studied the reaction progress by HPLC–MS (see Figure S2) and found a 98.0% and a 76.8% GPh reduction under the same conditions described vide supra. Degradation is confirmed by the appearance of reaction products in the NMR and MS spectra showing the presence of formyl glycine (F‐Gly), trough assigned signals (^13^C NMR in D_2_O: *δ* = 20.36 ppm; *δ* = 165.67 ppm; and *δ* = 176.68 ppm), HMBC correlation spectra, and the identification of an MS fragment of 101.94 g.mol^−1^ (FGly M.W. 101.02 g.mol^−1^) (see Figures S3, S4, and S10, Supporting Information). As Zr‐based MOFs are stable in water, we further probed catalysts reusability by recovering the crystalline powder after the first GPh degradation cycle and evaluating its performance in a consecutive reaction. We observed by ^1^H NMR that nMOF‐808 remains active with almost no loss of efficiency (≈91% GPh degradation within 2 h at room temperature, Figure S5, Supporting Information.

The postreaction MOFs were further recovered and digested for analysis, where a phosphonate compound different from GPh was observed (^31^P NMR in D_2_O: *δ* = 17.36 ppm) (see Figure S6, Supporting Information). Such compound is later identified as hydroxymethyl phosphonate in the mechanism proposed through synchrotron analysis on the postreaction recovered catalysts and discussed in subsequent sections [vide infra, mechanism Figure 6]. So far, the identification of FGly and the phosphonate by NMR and MS indicates that the only allowed pathway on the degradation reaction should follow the breaking of the C_β_─N bond (see Figure 1). These results suggest that methyl phosphonate and glycine are generated as intermediate products that continued to react to finally produce F‐Gly and the phosphonate that remains attached to the MOF. The degradation results were attributed specifically to the MOF activity as stability experiments of GPh in D_2_O showed no changes in the spectra after 7 days. Reactions in dark conditions and diffuse reflectance UV–Vis (DRUVS) spectra of MOFs allow to discard photocatalytic effects from ambient light (Figure S7). GPh degradation of MOFs is also compared with the Zr‐oxo clusters (nMOF‐808 synthesis Supporting Information, 11.9% degradation Figure S8), indicating that the connected structure of MOFs offers catalytic advantages over separated individual metal sites. It is important to note that the observed difference between both MOFs reactivities cannot be attributed to the chemical structure, topology, or pore affinity given that changes in crystallite size does not significantly alter these parameters. As such, we conclude that the difference can be attributed to the higher number of unsaturated coordinative sites in the crystal structure of nMOF‐808, which also behave as active sites for this catalytic process [vide infra, thermogravimetric analysis (TGA, Figure S9, Supporting Information), PDF, and EXAFS fitting].

Synchrotron radiation characterization techniques were then used to understand the coordination of chemical species around the Zr‐SBU to help elucidate GPh degradation mechanisms and the differences in reactivity between MOFs of differing crystallite sizes. These analyses were developed using XAS, FT‐IR, and total scattering studies, with XAS measurements probing all possible elements of relevance in GPh fragmentation (C, O, P, and Zr). A total description of the species involved in all the materials used for the synthesis of MOFs and reactions with GPh is presented in Tables S3 and S4. As a reference, the postreaction materials are denominated MOF‐808‐GPh and nMOF‐808‐GPh for the studied heterogeneous catalysts.

**Figure 4:**
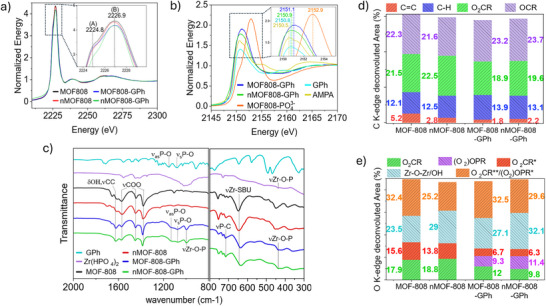
XAS of a) Zr L_3_‐edge for MOFs pre‐ and postreaction. b) P K‐edge for MOFs postreaction compared with standards aminomethyl phosphonic acid (AMPA), glyphosate GPh, and coordinated phosphoric acid MOF‐808‐PO_4_^3−^. See peak intensities of Figure 4a,b in Table S5. c) FT‐IR spectra of MOFs pre‐ and postreaction compared to GPh and Zr(HPO_4_)_2_ standards (range below 800 cm^−1^ was measured with synchrotron radiation). Deconvolution analysis of MOFs pre‐ and postreaction for d) C K‐edge and e) O K‐edge with calculated area % of functional groups. For *, **, and deconvolution analyses please refer to Figures S12–S15.

The local Zr environment was first probed using the Zr L_3_‐edge as this transition is highly sensitive to local symmetry and polarization of Zr atoms.^[^ ^53^ ^]^ All evaluated MOFs displayed two peaks (positions A and B), which are located at 2224.8 and 2226.9 eV, respectively, Figure 4a. The shape of the peaks and their energy difference (2.1 eV) indicates a coordination number (CN) of 8 that resembles the local structure of tetragonal ZrO_2_.^[^ ^54^ ^–‐^ ^56^ ^]^ These results suggest that the Zr coordination's sphere is saturated beyond the Zr cluster, likely incorporating molecular water, OH groups, carboxylates, and/or phosphonates. Pre‐ and postreacted MOFs featured this Zr coordination, indicating that direct Lewis's acid interactions of Zr‐SBU should occur after the exchange of terminal ligands linked in the MOF equatorial region, Figure 2. The reduction of peak intensity in the postreaction samples is likely due to the substitution of labile carboxylate groups (pK_a_ ∼ 4)^[^ ^57^ ^]^ with phosphonate groups that have a lower pK_a_ (pK_a_ ∼ 2.4),^[^ ^58^ ^]^ which displaces the charge density toward the Zr atoms that dictates the excitation of electrons from 2p orbitals to 3d empty states.^[^ ^59^ ^]^

P K‐edge analysis was then performed as it is highly sensitive to P speciation^[^ ^60^ ^]^ and thus useful in identifying the remaining phosphorous species trapped in the MOF post GPh degradation. Figure 4b compares the P K‐edge spectra of MOF‐808‐GPh and nMOF‐808‐GPh, along with reference materials of AMPA, GPh, and MOF‐808‐PO_4_^3−^. The latter of which is simply MOF‐808 loaded with phosphates obtained from degradation tests with *p*‐nitrophenyl phosphate (Figure S11). The analysis of the P species is completed by comparing the P K‐edge of MOFs with the reference materials; and the reported P spectra from the literature [see References (61, 62, 63, 64)] and the products from the degradation paths are presented in Figure 1. Differences in the shape, postedge features, and peak position of P K‐edge spectra allow us to eliminate the presence of P‐containing species from GPh, AMPA, and PO_4_^3−^, therefore validating the catalytic activity and discarding degradation paths a) and b) from Figure 1. Phosphates are discarded because of the peak gap of 1.8 eV observed between the postreaction materials and MOF‐808‐PO_4_^3−^.Meanwhile, GPh and AMPA display postedge features at 2158 and 2155 eV, respectively, with the latter showing a split peak, which is not distinguished in the postreacted MOFs (see further details in the Supporting Information). Such differences confirm the results observed with NMR, indicating that the trapped phosphorus product corresponds to a phosphonate obtained after the break of the C_β_─N bond of GPh. A gap of 0.2 eV is detected between the two postreacted MOFs, which can be attributed to the differences in the coordination around the Zr‐SBU on both crystal sizes as P K‐edge analysis is very sensitive to the surrounding environment of the P central atom and bond lengths, with larger distances shifting the peaks to lower energies.^[^ ^65^, ^66^ ^]^ This is the case for the P─O bonds of nMOF‐808‐GPh, which are larger than in coordination with the larger crystals (vide infra, PDF, and EXAFS fitting, Figures S16–S18, Supporting Information).

Synchrotron based FT‐IR was then performed to probe interactions in the coordinated ligands in the MOF caused by GPh degradation (Figure 4c). MOF‐808 IR spectra is broadly described with the regions 1300–1650 cm^−1^ assigned to BTC and carboxylate ligands, bands around 1100 cm^−1^ assigned to labile ligand, and two prominent bands at 644 and 450 cm^−1^ assigned to the coupling of Zr‐SBU with coordinated ligands.^[^ ^67^ ^]^ After the reaction, new bands are observed at 435, 716, 996, 1078, and 1121 cm^−1^. Absorption bands at 435 and 996 cm^−1^ are related to the metal complexation of Zr with phosphonates as compared, respectively, with Zr(HPO_4_)_2_ standard and reported DFT studies on the υ_s_ [P–(OFe)_2_] mode of bidentate P complexes with goethite.^[^ ^68^ ^]^ As such, IR results indicate a bidentate coordination of the phosphonate with Zr. In addition, characteristics peaks of Zr‐SBU are redshifted due to phosphonate–MOF interactions.^[^ ^36^ ^]^ Meanwhile, the peaks at 716, 1078, and 1121 cm^−1^ are attributed, respectively, to υ_s_ of P‐C,^[^ ^69^ ^]^ υ_s_ of P‐O and υ_as_ of P‐O^[^ ^34^ ^]^ of the trapped phosphonate.^[^ ^35^ ^]^ In addition, 1621 cm^−1^ peak increase is related to interactions of trapped molecules with BTC (δOH and νCC),^[^ ^70^ ^]^ whereas peak reduction and shifting at 1378, 1445, and 1574 cm^−1^, located in the region of carboxylate groups (υCOO^[^ ^31^, ^71^ ^]^), is attributed to the loss of formate ligands due to an exchange interaction with GPh.

The exchange of labile ligands like carboxylates was then explored using near‐edge X‐ray absorption spectroscopy (NEXAFs) at the C and O K‐edges, which allows the identification of functional groups at the surface of the crystals. Deconvolution analysis of C and O K‐edge was completed to further study the ligand coordination around the MOFs by discriminating the contributions from each chemical species. The analyses display a reduction in the peak areas at 288.4, 532.1, and 534.4 eV (peaks associated with coordinated carboxylates ─O_2_CR groups’, see Figure 4d,e), from the pristine catalysts to MOF‐808‐GPh and nMOF‐808‐GPh, respectively. The analysis also shows a new O peak contribution at 532.4 eV (associated with coordinated phosphonates‐(O_2_)OPR groups) for postreaction samples MOF‐808‐GPh and nMOF‐808‐GPh compared to the pristine MOFs samples, Figure 4e. Such reduction and the presence of a new peak contribution is associated to the substitution of terminal ligands in the MOF Zr‐SBU equatorial positions by the newly formed phosphonate, supporting the IR results. Furthermore, the area contribution of the O species at 536.5 eV (Zr‐O‐ZR/OH, associated with molecular water and hydroxyl groups, Figures 4e and S15) is higher in nMOF‐808  m, indicating more OH groups are present in nMOF‐808 compared with those in MOF‐808 before and after the reaction. This finding is directly related to the material's capacity for proton exchange and degradation rate.

Pair distribution function (PDF) analysis was then performed to better understand atomic scale structure through the Fourier transformation of high energy XRD patterns (HE‐XRD).^[^ ^72^, ^73^ ^]^ Importantly, PDF analysis can be performed on materials with limited crystallinity and/or chemical heterogeneity, which is especially informative here to assess the coordination of GPh reaction products to the Zr nodes. PDFs of the pristine MOFs (Figure S16a) display two representative peaks located at ≈2.2 and 3.5 Å, corresponding to Zr–O and Zr–Zr pair distances, respectively, whereas remaining peaks below 10 Å are related to the scattering of pairs in the Zr‐SBU. Peaks located at longer distances are related to the scattering contributions mainly from the Zr atoms with the organic ligands, as detailed in previous reports.^[^ ^74^, ^75^ ^]^ Broadly, the PDFs for nMOF‐808 are similar to MOF‐808, which is expected given similarities in periodic ordering shown in PXRD (Figure 3c). However, MOF‐808 presents less distortion at the Zr–Zr and Zr–O distances compared with nMOF‐808, as indicated with tighter FWHM, which indicates a comparative increase in ordering around the Zr‐SBU (Figure S16b). Broadening and shifting of peaks toward larger distances are observed in postreacted materials, explained by the retained phosphonate around the equatorial region of the Zr‐SBU (Figure S16c). The (O_2_)OPR group creates distortions in the PDFs at 2.2 and 3.5 Å distances because of the new Zr–O–P interactions. Such distortions can be expected due to the new pair contributions coming from P–O at (1∼2 Å) and Zr–P at (3∼4 Å), as well as changes in the Zr–O pairs at (2∼3 Å). These can be calculated in the differential PDFs (dPDF), by subtracting the pair contributions of the pristine MOFs from MOF‐808‐GPh and nMOF‐808‐GPh correspondingly. The dPDFs (Figure S16c) display two peaks common for both crystal sizes corresponding to P–O (≈1.5 Å) and Zr–P (≈3.6 Å) pairs, associated with the Zr–O–P coordination. In contrast, changes in the Zr–O pairs are not consistent, displaying different contributions attributed to variations in the Zr–O coordination sphere of MOFs after the reaction.

**Figure 5:**
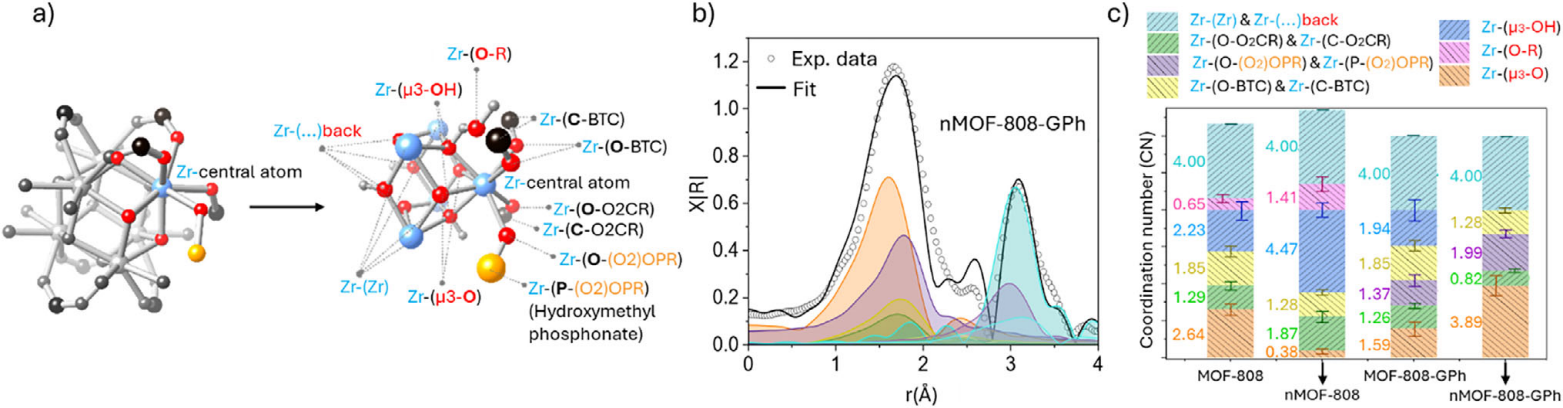
a) Description of scattering paths used in EXAFS refining. b) EXAFS fit of nMOF‐808‐GPh showing scattering paths contributions as described in a) (K weight 2, Δ*k* = 3.0–11.3 Å^−1^; Δ*R* = 1.0–4.0 Å). c) Comparison of CN for each scattering path as described in a), for MOFs before and after reaction (the color code for legends of scattering paths is the same in b) and c).

We then employed extended X‐ray absorption spectroscopy (EXAFS) at the Zr K‐edge to evaluate the local environments around the Zr node atoms. The local coordination environment of the Zr nodes is described in Figure 5a, which displays the scattering paths used in subsequent Zr EXAFS modeling. The scattering data obtained is Fourier transformed and modeled to account for the contribution of each path (Figure 5b).^[^ ^59^, ^76^, ^77^ ^]^ Such analysis reveals structural information in the short‐range limit such as the coordination numbers (CN) of the ligands around the Zr‐SBU. Changes in the CN of evaluated paths for the MOFs pre‐ and postreaction are used to confirm the presence or absence of different ligands [BTC, O_2_CR, (O_2_)OPR, OH, and OR (coordinated water or solvents)], providing insights into the degradation mechanism of GPh and catalytic activity differences in the two crystal sizes.

The EXAFS data of all pre‐ and postreacted MOFs display two distinctive peaks (Figures 5b, S17, and S18). The first peak (≈1.5 Å, not phase corrected) groups the scattering from the first coordination shell of O atoms, whereas the second peak (≈3.1 Å, not phase corrected) includes the scattering of the nearest 4 Zr atoms from the SBU, the C and P atoms from coordinated ligands, and the remaining O atoms in the SBU. The EXAFs data is fitted according to the previous description and the obtained CN for all samples are summarized in Figure 5c. All refined data details and models are included in Figures S17 and S18, Supporting Information. Although both chemical structures present in MOF‐808 and nMOF‐808 are the same, we observed differences in the presence of weakly coordinated species at the equatorial region (Figure 2), such as water and terminal ligands, which likely affect GPh degradation properties. The CN of the scattering paths Zr‐(μ3‐OH), Zr‐(O‐O_2_CR), and Zr−(μ3‐O‐R) for the nMOF‐808 are higher than MOF‐808 (CN 4.47 > 2.23, 1.87 > 1.29 and 1.41 > 0.65, respectively), whereas MOF‐808 display a higher CN for the Zr−(μ3‐O) (CN 0.38 < 2.64) (Figure 5c). Such differences indicate a higher presence of hydroxyl groups, formates, and other terminal ligands, coordinated to the labile equatorial positions of the Zr‐SBU in the nMOF‐808, which can be associated with a higher reactivity. At the same time, the CN of the path Zr−(O–BTC) is lower for the nMOF‐808 compared to MOF‐808 (CN 1.28 < 1.85), which also attributes to the presence of defects in the structure. Comparing the fitting results of pre to postreaction materials, respectively (e.g., nMOF‐808 versus nMOF‐808‐GPh), it can be observed how the CN of labile species from paths Zr‐(μ3‐OH) and Zr‐(O‐O_2_CR) (protonated oxygen and carboxylate ligands) are reduced in number, whereas a new contribution appears from the Zr−(O‐P) path, which confirms the presence of a ligand exchange mechanism associated with the substitution of OH and formates by phosphonate groups.

**Figure 6:**
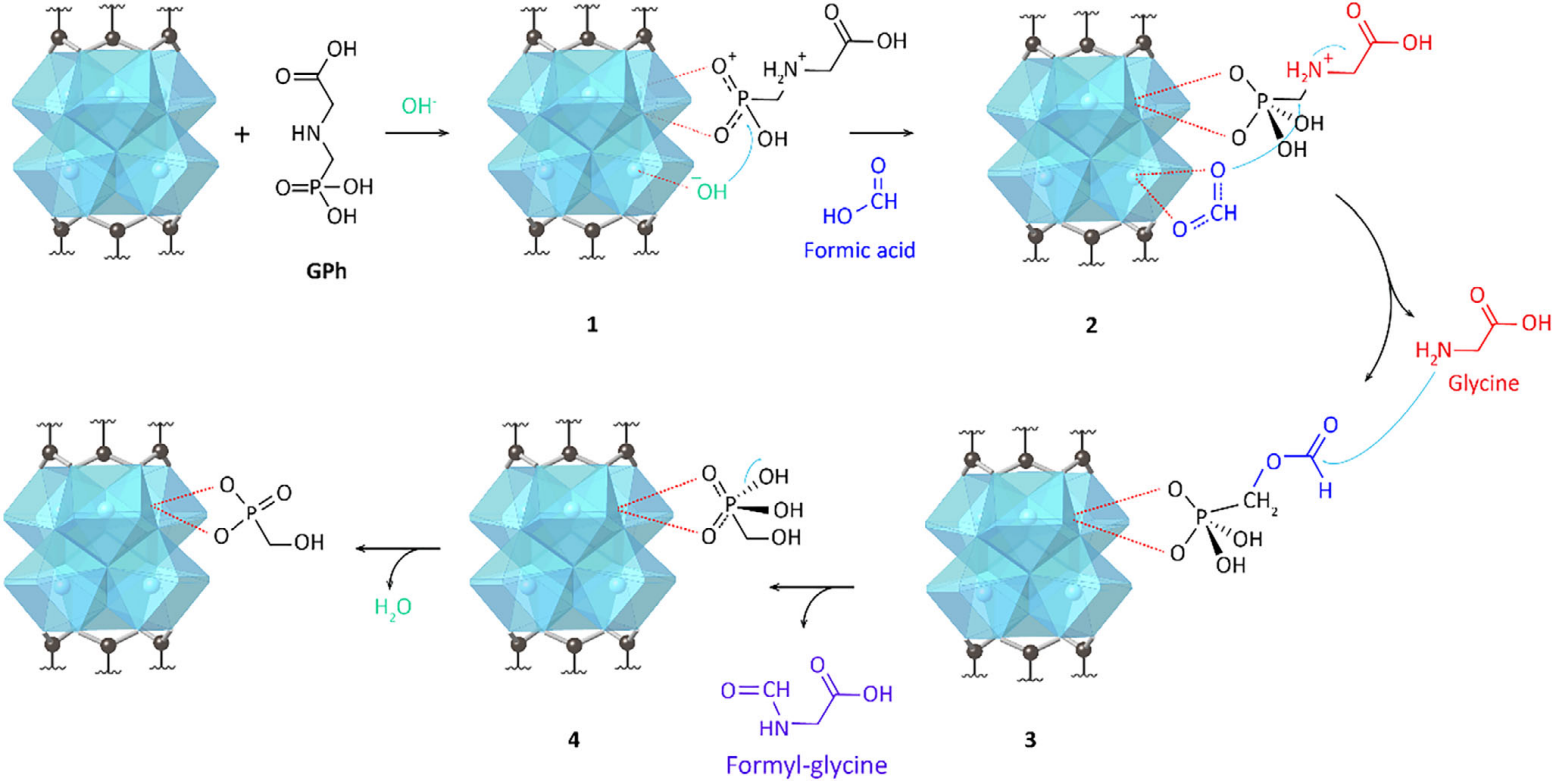
Proposed degradation mechanism for GPh using MOF‐808 as a heterogeneous catalyst.

Our characterization efforts detailed above help fully elucidate the reaction mechanism of GPh on MOF‐808, and further explain the influence of undercoordinated Zr sites in reactivity. The Zr‐L_3_‐edge spectroscopy analysis indicates that coordination of phosphonate groups is completed by ligand exchange, whereas IR and K‐edge XAS of C, O, and Zr further indicates a reduction in carboxylate groups for the MOFs postreaction (Figures 4 and 5). These conclusions strongly suggest that adsorbed GPh can remove monocarboxylate ligands from the MOF‐808 equatorial region by ligand exchange before starting the degradation reaction. Ligand exchange would be expected to lead to a bidentate bridging bonding as it is more energetically favored than creating a chelating complex,^[^ ^43^ ^]^ which is also observed on the IR band at 996 cm^−1^ (Figure 4c). Such effects create a charge displacement toward the Zr‐SBU and the other carboxylate ligands, promoting further ligand exchange in the other locations of MOF‐808 while preserving the integrity of the Zr‐SBU.^[^ ^78^ ^]^ Given these findings, the mechanism of GPh degradation is summarized in Figure 6. According to this mechanism, GPh degradation begins with a ligand exchange reaction where the formates coordinated to the equatorial positions of the Zr‐SBU are replaced by the phosphonate group in GPh (1, Figure 6). In parallel, hydroxyl groups coordinated to the Zr‐SBU help to stabilize GPh by creating a P penta‐coordination, similar to reported mechanisms of OPC interacting with Zr‐MOFs.^[^ ^43^ ^]^ This facilitates the nucleophilic attack of formates in solution on the C_β_, leading to C─N bond breaking to release Gly and leaving coordinated to the SBU a formyl ester moiety (3, Figure 6). In the following reaction step, the amine group from the Gly behaves as a nucleophile reacting with the electrophilic carbon of this formyl ester (4, Figure 6). Finally, F‐Gly is released while leaving hydroxymethyl‐phosphonate attached to the Zr‐SBU. As formates and hydroxyl groups are determinant to initiate this mechanism, the more O_2_CR and OH^−^ groups coordinated to the SBU, the better. Such is confirmed by the improvement in the catalytic performance of Zr_6_‐oxoclusters and MOFs in the presence of HCOOH (Figures S8 and S19). Additionally, digestion of postreacted MOFs display significant amounts of formates providing a constant supply for multiple degradation cycles (Figures S20b and S21b). The digestion of postreacted MOFs also confirms that phosphonate products remain trapped in the MOF structure (Figures S20c and S21c). However, there are not enough metal active sites for all hydroxymethyl phosphonate to remain coordinated to the Zr‐SBU in the conditions evaluated. Considering that the interaction of phosphonates in excess with Zr‐based MOFs force the displacement of ligands^[^ ^79^ ^]^ while increasing the acidity of Bronsted acid sites,^[^ ^80^ ^]^ it can be thought of phosphonates being displaced among themselves. As no MOF degradation is observed according to total diffraction data (PDF analysis, Figure S16) and NMR data (Figure S1, absence of *δ* = 8.39 ppm, BTC), the produced hydroxymethyl phosphonate should be exchanged by incoming GPh while remaining trapped in the MOF porous structure due to the more hydrophilic pore environment, as indicated by the increase of BTC FT‐IR bands of postreacted MOFs at 1621 cm^−1^ (Figure 4c). Furthermore, by comparing both crystal sizes, experimental results indicate that nMOF‐808 possesses more coordinatively unsaturated sites, as reflected in a higher presence of such labile ligands (C and O K‐edge NEXAFS, Figures S13b and S15b, changes at 288.4 and 536.5 eV peak area contributions, associated with O_2_CR and OH groups, respectively), as well as Zr EXAFS refining (Figure 5c, Zr‐(OR) and Zr‐(O‐O_2_CR) paths). Such configuration favors the exchange of reactive species in the degradation of GPh and accounts for the higher activity of smaller crystals.

## Conclusions

In this study, MOF‐808 was not only able to capture GPh, as other Zr‐based MOFs, but it can also degrade it, acting as a heterogeneous catalyst. Higher catalytic performance is observed on MOF‐808 smaller crystals (themed nMOF‐808) favored by a higher presence and consumption of labile ligands like formates and hydroxyl groups. Such a reaction led to the breaking of the C_β_─N bond of GPh producing F‐Gly. To the best of our knowledge, this reaction has not been reported earlier and represents an important contribution to the final disposal of a large excess of dangerous GPh applied to farms around the globe. The degradation pathway we report in this work, takes place in mild conditions and relatively short time. Finally, we could observe that the nMOF‐808 can be reused, and that the potentially toxic by‐product of this GPh degradation pathway (hydroxymethyl‐phosphonate) remains strongly coordinated to the MOF, therefore eliminating the possibility of poisoning the environment.

## Conflict of Interests

The authors declare no conflict of interest.

## Supporting information



Supporting Information

## Data Availability

The data that support the findings of this study are available from the corresponding author upon reasonable request.
