# Supplementary material for: Degradation of Glyphosate to Benign N‐Formyl Glycine Using MOF‐808 Nanocrystals
Source: Angew Chem Int Ed Engl. 2025 Mar 17;64(21):e202424540. doi: 10.1002/anie.202424540 (PMC12087855; doi:10.1002/anie.202424540)
Supplement: Supplementary file 1 — Supporting Information [file ANIE-64-e202424540-s001.docx]

Degradation of Glyphosate to Benign N-Formyl Glycine Using MOF-808 Nanocrystals

Jhair A. Peña Prada^†^, Tatiana A. Huertas Navarro^†^, Stephanie L. Chua, Alejandro M. Granados, Chih-Wen Pao, Alejandro M. Fracaroli*, Nicholas M. Bedford*

[*] J.A. Peña, S. Chua, N.M. Bedford
School of Chemical Engineering
The University of New South Wales
Sydney, NSW 2052, Australia
E-mail: [n.bedford@unsw.edu.au](mailto:n.bedford@unsw.edu.au)

N.M. Bedford
 Department of Chemistry
 Colorado School of Mines
 Golden, CO 80401, United States
 E-mail: [nicholas.bedford@mines.edu](mailto:nicholas.bedford@mines.edu)

T.A. Huertas, A.M. Granados, A.M. Fracaroli

Departamento de Química Orgánica, Facultad de Ciencias Químicas
Consejo Nacional de Investigaciones Científicas y Técnicas (CONICET)

Instituto de Investigaciones en Fisicoquímica de Córdoba (INFIQC)

Universidad Nacional de Córdoba
X5000HUA, Córdoba, Argentina

E-mail: [a.fracaroli@unc.edu.ar](mailto:a.fracaroli@unc.edu.ar)

C.W.Pao
National Synchrotron Radiation Research Center
Hsinchu 30076, Taiwan

[^†^] These authors contributed equal to this work

# Supporting Information

[Supporting Information 1](#_Toc190267503)

[Materials and Methods 2](#_Toc190267504)

[Reagents 2](#_Toc190267505)

[MOF-808 synthesis 2](#_Toc190267506)

[nMOF-808 synthesis 2](#_Toc190267507)

[Pristine MOFs characterization 2](#_Toc190267508)

[Glyphosate degradation experiments 3](#_Toc190267509)

[Synchrotron measurements. 3](#_Toc190267510)

[Data analysis 3](#_Toc190267511)

[P K-edge and phosphorous speciation analysis. 3](#_Toc190267512)

[C K-edge deconvolution analysis 4](#_Toc190267513)

[O K-edge deconvolution analysis 4](#_Toc190267514)

[Pair distribution function PDF analysis 4](#_Toc190267515)

[EXAFS modelling of pristine and after-reaction samples 5](#_Toc190267516)

[NMR, HPLC-MS and TGA data: (Fig. S1 - Fig. S11): 10](#_Toc190267517)

[Synchrotron data (XAS and total scattering): (Fig. S11 - S18) 20](#_Toc190267518)

[Experiments with formic acid (Fig. S19 – S21) 28](#_Toc190267519)

[References 31](#_Toc190267520)

# Materials and Methods

Reagents. N,N-dimethylformamide (DMF, purity>98 %), zirconyl oxychloride octahydrate (ZrOCl_2_∙8H_2_O, purity > 98 %), zirconium chloride (ZrCl_4_, purity > 98 %), 1,3,5−benzenetricarboxylic acid (trimesic acid, BTC), formic acid (HCOOH), acetic acid (CH_3_COOH), ethanol (EtOH) and acetone (CH_3_)_2_CO, glyphosate (GPh), deuterated water (D_2_O), were all purchased from Sigma Aldrich (Merck) and used as received.

MOF-808 synthesis (crystal size ~500 nm side) was prepared using a solvothermal method adapted from the literature (see ref^[1]^). In a typical synthetic procedure, two separated 20 mL scintillation vials were prepared. In the first vial, 0.16 g (0.50 mmol) of ZrOCl_2_.8H_2_O were added together with 10 mL of distilled N,N−dimethylfomamide (DMF). In the second vial, a solution of 0.11 g (0.52 mmol) of benzene-1,3,5-tricarboxylic acid (BTC) and 10 mL of DMF. Later, 20 mL of formic acid 98% was added as modulator to the first vial containing the Zr precursor. This addition was followed by the mixing of both 20 mL vial solutions and sonication for 5-10 min. After obtaining a clear transparent solution, the 20 mL scintillation vial was capped and heated at 130°C for 3 days. Subsequently, white crystals were recovered after cooling down the suspension, by centrifugation at 7000 rpm for 3 min. After the centrifugation, the supernatant was discarded and the remaining solid was re-suspended in 5 mL of fresh DMF and then vortexed and centrifuged again. This whole procedure (centrifuge-decantation-fresh solvent addition) was repeated 3 times a day with fresh DMF, and 3 more times a day with methanol (MeOH). The remaining solid was dried under vacuum and room temp. for 3 h. Finally, 0.16 g (0.12 mmol) of a white crystalline power was recovered by low-pressure filtration, corresponding to a 23.5% reaction yield (based on the Zr precursor added and the molecular formula of MOF-808, C_24_H_16_O_32_Zr_6_).

nMOF-808 synthesis (crystal size ~65 nm side) was obtained by a similar two-step procedure to the one reported recently (see ^[2]^). In a 20 mL vial, 2.0 g (8.58 mmol) of ZrCl_4_ was added to a mixture of 3 mL of acetic acid and 5 mL of isopropanol (iPrOH), under constant stirring. Then, the suspension was heated at 120 °C for 60 min. After this reaction, the Zr_6_(µ_3_−O)_4_(µ_3_-OH)_4_(CH_3_COO-)_6_ (or simply Zr_6_−oxoclusters) are recovered by centrifugation at 9000 rpm for 5 min., washed with acetone (2 times a day, each of them followed by centrifugation and vortex). After the last washing, the whitish crystalline powder was filtered at low pressure and dried under dynamic vacuum at room temp. for 3 h. After this “activation” procedure 1.75 g (1.72 mmol) of Zr6−oxoclusters were obtained (20.1% reaction yield based on Zr precursor).

In the second reaction step, 0.60 g (0.59 mmol) of these Zr_6_−oxoclusters were added as white powder to a different 20 mL scintillation vial, containing 0.75 mL of concentrated formic acid solution (98%) and 1.25 mL of H2O. The mixture was stirred until the complete dissolution of the solid precursor (forming a clear transparent solution). After this, 0.150 g (0.7 mmol) of BTC were added (mass ratio 4:1 with respect to Zr precursor), and the suspension was stirred overnight at room temp. The obtained gelified whitish suspension of nMOF-808 crystals were recovered by centrifugation (9000 rpm for 5 min.), washed with 55 mL of a mixture of H2O/acetone (0.8:1.0) twice in a day. The white solid was collected by low-pressure filtration and dried under dynamic vacuum and room temp. This methodology allowed us to obtain 0.150 g (0.11 mmol) of nMOF-808 as a white crystalline powder (18.6 % yield, with respect to added Zr_6_−oxoclusters).

Pristine MOFs characterization. The solvothermal reactions were carried out in a Thermo Scientific - HERATHERM OGH60 oven, and the resulting materials were activated by washing the crystalline powder using a centrifuge Eppendorf 5804. Then, the characterization was performed by Scanning Electron Microscopy (FE-SEM Carls Zeiss Σigma), nitrogen adsorption experiments (Micromeritics ASAP2020), powder X-ray diffraction (Malvern Panalytical X’Pert Pro), thermogravimetric analysis (TGA, Discovery).

Glyphosate degradation experiments. These experiments were performed in a 20 mL scintillation vial using deuterated water (D_2_O) as solvent, and with constant stirring at room temperature. The reaction progress was followed by nuclear magnetic resonance (using a Bruker Ultrashield 400 MHz Advance NEO) and by HLPC-Ms (Waters Acquity UPLC H-Class instrument, equipped with an Agilent C-18 SB-AQ (2,1 x 150) mm column with particle size of 3,5 µm, and electrospray ionization detection method.

Synchrotron measurements. Samples were prepared from powders of pristine MOFs and post-reacted recovered materials after centrifugation and vacuum activation. ***NEXAFS (C and O K-edges)*** - Powders were placed on C tape and measured at SXR beamline at the Australian Synchrotron. Data was collected from total electron yield (TEY, surface measurement) and calibrated with C and O standard measured in parallel for each sample. Data was processed using QANT software^[3]^. The absorption intensity and the transition energies values obtained in the areas of the deconvolution analysis of C and O K-edge are proportional to the relative concentration of functional groups and are used as a semi-quantitative tool for comparison among the samples (see Figs. S12-S15). ***XAS (Zr L-edge and P K-edge)*** - Powders were placed on C tape and measured at MEX-2 beamline at the Australian Synchrotron, data was collected in fluorescence mode and processed using Athena software^[4]^. ***IR:*** The materials were measured as pure powders using the ATR accessory at the THz beamline at the Australian synchrotron to look up for the region below 800 cm-1. Data was processed using OPUS spectroscopy software from Bruker. ***XAS (Zr K-edge):*** Data was measured as pure powders placed on capillary Kapton tubes at the Taiwan Light source (TLS) synchrotron. Data was collected and processed in Athena and refined in Artemis software (K weight 2; Δk = 3.0-11.3 Å^-1^; ΔR = 1.0-4.0 Å)^[4]^. ***Total scattering (PDF analysis):*** Data was measured as pure powders placed on capillary Kapton tubes at the Deutsches Elektronen Synchrotron (DESY) using radiation of λ= 0.1223 Å. Data was processed using PDFgetx3 software^[5]^ to q_max_ 27. Data was normalized using origin software.

## Data analysis

P K-edge and phosphorous speciation analysis. Reference sample MOF-808-PO_4_^3-^ was obtained from the degradation tests with p-nitrophenylphosphate (pNPP), whose only reaction products are phosphoric acid and p-nitrophneol (pNP) ^[6–8]^. Test reactions of pNPP with MOF-808 were completed using mol ratio pNPP/MOF=10, and reaction products were followed using Uv-Vis and P K-edge, Fig S11. The peak gap of 1.8 eV observed between MOF-808-PO_4_^3-^ and post-reaction materials indicate that phosphates are not the product of the reaction. The GPh and AMPA standards from Fig. 4b display a different peak shape, and extra peak features at 2158 and 2155 respectively, compared to the post-reaction MOFs, indicating that attached phosphorous structures differ from GPh and AMPA. Extra peak features of GPh and AMPA can be related to the scattering of lone pair electrons from N atoms which would not be present in the obtained phosphonate product attached to MOF-808.

Fig. 1 shows the possible reaction of phosphorus products that can be attached to the structure of the MOF-808 after the degradation of GPh according to the cleavage of the C-C, Cα-N, and Cβ-P bonds. Beyond the comparison with standards, the path leading to AMPA would require the direct coordination of the N lone pairs with unsaturated $\text{Zr}_{\text{6}}^{\text{4+}}$ ^[9],^ which according to the results of Zr L3-edge would not be expected since $\text{Zr}_{\text{6}}^{\text{4+}}$ is saturated with 8 oxygens in the first coordination shell before and after reaction, indicating that ligand exchange is only permitted trough oxygen atoms. Besides, GPh degradation experiments with fluorine-doped BiVO_4_ (another tetravalent metal oxide) showed that oxygen-saturated metal oxides prevent the generation of AMPA ^[10,11]^. The Sarcosine Path will produce phosphoric acid ^[12,13]^ that according to the peak position of the reference MOF-808-PO_4_^3-^ does not correspond with the post-reaction materials Fig 4b. Furthermore, protonated species of PO_4_^3-^ display peak shifts in the range of 0.3 eV which is a short gap compared to the peak position difference of after-reaction MOFs samples in Fig 4b and MOF-808-PO_4_^3-^ (>1.8 eV) ^[14]^. Breaking the C-C bond in the structure will lead to the production of CO_2_ and no other organic products ^[15]^, which does not match with the NMR results obtained. Furthermore, it was not observed bubbles during the reaction. The remaining degradation route follows the breaking of the C_β_-N bond leading to the production of methyl-phosphonate and glycine, which react further with monocarboxylate ligands and solvents to produce formyl-glycine and attached hydroxymethyl-phosphonate as explained in reaction mechanism (vide infra).

C K-edge deconvolution analysis (Figs. S12-S13) C species involved in the before and after reaction samples are assigned according to the C species used in the materials for the MOF synthesis and the reactions with GPh (Tables S3 and S4), the results obtained from reference samples (GPh, BTC and Zr-oxo, Fig. S12b), and peak positions of functional groups reported in the literature ^[16–20]^. Four main peaks associated with 1s-π* and 1s-σ* transitions are assigned as presented in Fig. S13. C K-edge spectra of the reactions with regular size MOF-808 and deconvolution graphics of all samples are presented in Fig. S13a.

The deconvolution analysis was completed using the software QANT ^[3]^ developed at the Australian synchrotron. The peaks around 284.8-285.3 are assigned to the C=C 1s-π* transition of the aromatic rings from the BTC ligand(Fig S12b and Table 1 of ^[17]^). The pristine MOFs (MOF-808 and nMOF-808) display a peak at 284.8 eV, while the after-reaction (MOFs-GPh) materials display a shift to 285.3 eV associated with changes in the local environment of the ligand after accommodating the reactive species. The peak at 287.5 is assigned to C-H 1s-σ* transitions from methyl groups (Fig S12b, Fig 3 of ^[19]^, and Fig 4 of ^[18]^), associated with organic solvents and monocarboxylate ligands molecules (acetic and formic acid) which can be accommodated in the defect sites, monodentate coordinated with the oxygens from the Zr_6_(µ_3_-O)_4_(µ_3_-OH)_4_ cluster or coordinating with monocarboxylate ligands of the free equatorial locations of the MOF-808 structure ^[21]^. The main feature of the absorption peaks is located at 288.4 eV corresponding to O_2_CR 1s-σ* transition of the carboxylate groups from the BTC ligands that connect the Zr clusters and from monocarboxylate terminal ligands in the MOF-808 equatorial region (Fig S12b, Table 1 of ^[17]^, and Fig 4 of ^[19]^). The peaks at 289.5 eV correspond to the 1s-σ* transition of OCR groups of solvents coordinating the in the MOF (FigS12b and Fig 4 of ^[18]^). Finally, 290.1 peaks are related to multiple scattering effects that occur close to the ionization energy which is defined around 290 eV (page 1057 of ^[17]^, page 14083 of ^[16]^, and Fig 7 of ^[20]^) and indicated by the step function in Fig. S13a.

O K-edge deconvolution analysis (Fig. S14-S15) Deconvolution of O K-edge analysis is made, similarly to the C K-edge analysis with peaks assigned according to the list of O species present in the materials (Tables S3 and S4), the comparison with standards (GPh, BTC, and ZrO_2_, Fig. S14b), and functional groups reported in the literature ^[22–26]^. Five primary absorption peaks are used to identify the oxygen species involved in the reactions. O K-edge spectra of the reactions with regular size MOF-808 and deconvolution graphics of all samples are presented in Fig. S15.

The peaks of 532.1, 534.4, and 539.6 correspond to the 1s-π* and 1s-σ* transitions of the carboxylate groups associated with the BTC ligand and the coordinated monocarboxylate ligands in the equatorial region of the MOF-808 (FigS14b, Fig 20 of ^[22]^ and Table 1 of ^[26]^). The peak at 532.4 eV corresponds to the contribution of phosphate group 1s-π* transitions from phosphonate reaction products (FigS14b and Fig 11 of ^[25]^). The phosphonate groups also overlap its 1s-σ* transition at 539.6 eV with the contributions of carboxylates (FigS14b, Fig 1 of ^[23]^ and Fig 11 of ^[25]^). The peak at 536.5 is assigned to Zr-O-Zr 1s-σ* and molecular water 1s-σ* transitions from the Zr_6_(µ_3_-O)_4_(µ_3_-OH)_4_ cluster (FigS14b and Fig 45 of ^[22]^). Finally, the peak at 541.3 corresponds to multiple scattering effects and is limited by the step function placed at around 540 eV that marks the ionization energies of C=O and OH functional groups as reported in the literature ((FigS14b, ^[24]^and Table 1 of ^[26]^)

Pair distribution function PDF analysis (Fig. S16) Total scattering data is processed from Q space using PDFgetx3 to obtain the corresponding G(r) functions (PDF analysis) using a q_max_ of 27. PDFs are then normalized to 1 with the highest intensity (Zr-Zr peak) since no significant changes would be expected in the Zr-SBU integrity by the reaction with GPh; this peak can be used as a reference. Differentials are obtained by subtracting pair contributions from the pristine MOFs to the after-reaction materials. The differentials are normalized again to identify the peaks from the coordination of GPh with MOF-808.

EXAFS modelling of pristine and after-reaction samples. FT-EXAFS data was obtained using Athena software and modelling was completed using Artemis software. The modelling requires the description of the scattering paths, which are obtained from the interactions of the central Zr atom with the SBU Zr-(µ_3_-**O**), Zr-(µ_3_-**O**H), Zr-(µ_3_-**O**)2(µ_3_-**O**H)2 back, Zr-Zr; the BTC linkers Zr-(**O**-BTC), Zr−(**C**-BTC); the compounds R (water or solvents) coordinated to the cluster (Zr-(**O**-R); the monocarboxylate ligands attached in the equatorial region [Zr−(**O**-O_2_CR), Zr-(**C**-O_2_CR)]; and the coordinated phosphonate species Zr-(**O**-(O_2_)OPR) and Zr-(**P**-(O_2_)OPR) (the scattering atom in each path is in bold text). The distances used for the model were obtained from the reported MOF-808 CIF file [see ref. ^[1]^], the calculated differential PDFs (Fig. S16c) and reported DFT models^[27]^. The analysis of this Zr coordination sphere, is divided as depicted in Fig. 5a, as each path is being calculated with the corresponding coordination number (CN).

(Figs. S17-S18). Every Zr atom displays a different environment since monocarboxylate ligands and phosphonate species are coordinating at different locations in the Zr-SBU then the results observed for the EXAFS fitting correspond to the average of the presence of the listed scattering paths. The EXAFS model calculates the coordination numbers of every scattering path multiplying a fixed common amplitude (0.784 obtained from Zr reference foil) times the variables denominated n (CN=amp*n) which are restricted to values according to the geometry of the Zr-SBU and the assumption of 8 coordination environment from the Zr L3-edge results. A total of 5 constants (variables “n” and “p”, v.i.) are used in the simulation. ΔR is calculated using the path distances multiplied by a factor alpha assigned per element in each path. Alpha1 for O, alpha2 for Zr, and alpha3 for C. Debye-Waller factors are assigned per element in each path. SS1 for O, SS2 for Zr, and SS3 for C. Including E0 it was used up to 12 parameters in the fitting out of around 18 independent variables for the range of data used (K weight 2, Δk = 3.0-11.3 Å-1; ΔR = 1.0-4.0 Å).

Ideally, the Zr-(µ_3_-O)_2_, Zr-(µ_3_-OH)_2_ paths should sump up together CN=4 corresponding to the 4 oxygen of the first coordination shell of each Zr-central atom with the Zr-SBU. Any positive difference is assigned to OH located at places without coordinated ligands. Therefore, the CN of Zr-(µ_3_-OH) is used to indicate the presence of protonated oxygen in the first coordination shell of each Zr atom, e.g. for MOF-808 Zr-(µ_3_-O) CN=2.64 and Zr-(µ_3_-OH) CN=1.85 (Fig 5c), adding them gives 4.49, the difference of 0.49 is assigned to OH that are located at defects or missing monocarboxylate ligands which are populated with OH and water molecules from the environment. On the other side, the paths Zr-(µ_3_-O)_2_(µ_3_-OH)_2_back and Zr-Zr also from the Zr-SBU are kept constant and fixed to CN=4 since the SBU is not expected to disintegrate during the reaction, see Fig 5a. The path Zr-(µ_3_-O)_2_(µ_3_-OH)_2_back considers an average distance of 3.886 Å to reduce the number of parameters in the model without affecting the analysis of coordinating species, Figs. S17 and S18. The CN of Zr-(O-R) is calculated using another variable “p” as a percentage of the CN of Zr-(µ_3_-OH), since R (water molecules or monodentate solvents) are attached through the OH groups.

Every Zr is attached to 2 carbons from organic linkers BTC, but this number might change due to defects in the structure. Then, the CN of BTC for the paths Zr-(C-BTC) and Zr-(O-BTC) must be the same and should be between 0-2 according to Fig 5a. The Zr-(C-BTC) and Zr-(O-BTC) of after-reaction samples are fixed taking the values found for the pristine MOF fittings since MOF degradation is not observed for the non-carbonated samples. Assuming a fully Zr−SBU saturated with bidentate bridging carboxylates in the equatorial region, there would be 2 carbons from monocarboxylate ligands (acetic acid, and formic acid) but this number might change due to the substitution of these groups by –OH, H2O, and phosphonate species during the reaction. Then, the CN of monocarboxylate ligands for the paths Zr-(O-O_2_CR) and Zr-(C- O_2_CR) must be the same and should be between 0-2 according to Fig 5a. Similarly, the CN of Zr-(O-(O_2_)OPR) and Zr-(P-(O_2_)OPR) should be the same and between 0-2 corresponding to the ligand exchange of labile species in the MOF (monocarboxylate ligands, OH- and H_2_O).

The model used a combination of variables (“n” and “p”) to calculate the coordination numbers (CN) of the scattering paths. The CN are restricted for the paths according to the geometry of the Zr-SBU and the possibility of coordination with organic ligands BTC and labile species on the free equatorial region. ΔR was calculated by multiplying the reference distance of each scattering path by an α factor per element. The Debye-Waller factors SS are also assigned per element. The set of equations used as inputs in Artemis are presented in Tables S1 and S2.

**Table S1**. Input parameters for each scattering path.

| Path | CN | CN restricted range | ΔR | SS |
| --- | --- | --- | --- | --- |
| Zr-(µ_3_-O) | CN1=abs(n1*amp) | 0-4 | reff*α1 | SS1 |
| Zr-(O-O_2_CR) | CN2=abs(n2*amp) | 0-2 | reff*α1 | SS1 |
| Zr-(O-(O_2_)OPR) | CN5=abs(n5*amp) | 0-2 | reff*α1 | SS1 |
| Zr-(O-BTC) | CN3=abs(n3*amp) | 0-2 | reff*α1 | SS1 |
| Zr-(µ_3_-OH) | CN4=8-CN1-CN2-CN3-CN5 | 0-4 | reff*α1 | SS1 |
| Zr-(O-R) | CN6=p*CN4 | 0-4 | reff*α1 | SS1 |
| Zr-(C-BTC) | CN3=abs(n3*amp) | 0-2 | reff*α2 | SS2 |
| Zr-(C-O_2_CR) | CN2=abs(n2*amp) | 0-2 | reff*α2 | SS2 |
| Zr-(Zr) | Fixed to 4 | - | reff*α3 | SS3 |
| Zr-(P-(O_2_)OPR) | CN5=abs(n5*amp) | 0-2 | reff*α2 | SS2 |
| Zr-(µ_3_-O)_2_(µ_3_-OH)_2_back | Fixed to 4 | - | reff*α1 | SS1 |

**Table S2**. Input parameters for each sample (v parameter used).

| Parameters | Materials | | | |
| --- | --- | --- | --- | --- |
|  | MOF-808 | nMOF-808 | MOF-808-GPh | nMOF-808-GPh |
| amp | Fixed | Fixed | Fixed | Fixed |
| E0 | v | v | v | v |
| n1 | v | v | v | v |
| n2 | v | v | v | v |
| n3 | v | v | Fixed | Fixed |
| n4 | calculated | calculated | calculated | calculated |
| n5 | - | - | v | v |
| p | v | v | v | v |
| α1 | v | v | v | v |
| α2 | v | v | v | v |
| α3 | v | v | v | v |
| SS1 | v | v | v | v |
| SS2 | v | v | v | v |
| SS3 | v | v | v | v |
| Total parameters used | 11 | 11 | 11 | 11 |

Statistics of the reported parameters are calculated by the software for ΔR and the Debye-Waller factors. The statistics for CN4 are calculated using equation (1), the CNs (1, 2, 3, 5) using equation (2), and CN6 using equation (3).

$$\delta CN4=\sqrt{{\delta CN1}^{2}+{\delta CN2}^{2}+{\delta CN3}^{2}+{\delta CN5}^{2}} (1)$$

$${\delta CN}_{i}={CN}_{i}*\sqrt{\left( \frac{\delta n_{i}}{n_{i}} \right)^{2}+\left( \frac{\delta amp}{amp} \right)^{2}} \left( 2 \right)$$

$$\delta CN6=CN6*\sqrt{\left( \frac{\delta CN4}{CN4} \right)^{2}+\left( \frac{\delta p}{p} \right)^{2}} (3)$$

**Table S3**. **C and O species of reactants** involved in the synthesis of MOF before reaction.

| **Materials for MOF Synthesis** | **Structure** | **C-species** | **O-species** |
| --- | --- | --- | --- |
| Acetic acid (nMOF-808 only) |  | Carboxylate-methyl | C=O, C-O-H |
| Isopropanol (nMOF-808 only) |  | C_2_COH-methyl | C-O-H |
| Formic acid |  | Carboxylate | C=O, C-O-H |
| Methanol | CH_3_OH | C-OH | C-O-H |
| Acetone | \|  \| \| --- \| | C_2_C=O-methyl | C=O, C-O-H |
| N,N-Dimethylformamide (MOF−808 only) |  | HNC=O-methyl | C=O |

**Table S4**. **C, O, P, Zr species of reactants** related to the MOFs materials after reaction.

| **Materials after Reaction** | **Structure** | **C-species (C-Sp)** | **O-Sp** | **P-Sp** | **Zr-Sp** |
| --- | --- | --- | --- | --- | --- |
| Water |  | - | H_2_O | - | - |
| GPh |  | P-C-N, N-C-C, Carboxylate | C=O, C-OH, P=O, P-OH | R(OH)_2_P=O | - |
| Organophosphonates  (GPh deg products) |  | R | P=O, P−OH, R | R(OH)_2_P=O | - |
| MOF-808 | 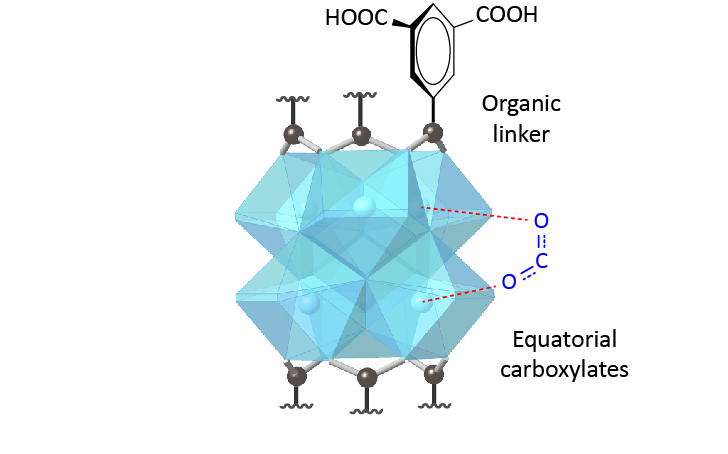 | Carboxylate, Aromatic | (Zr_3_)-O, Zr−O-C, Zr−O=C | - | Zr-O_8_ |

**Table S5.** Peak intensities of the **Zr L3-edge and P K-edge** analysis as presented in Fig 4.

| Sample | Zr L3 edge | | P K edge |
| --- | --- | --- | --- |
|  | I (A) | I (B) | I |
| MOF-808 | 3.27 | 4.37 | - |
| nMOF-808 | 3.14 | 4.33 | - |
| MOF-808-GPh | 3.19 | 4.19 | 1.91 |
| nMOF-808-GPh | 2.81 | 4.2 | 1.79 |
| AMPA | - | - | 1.61 |
| GPh | - | - | 1.41 |
| MOF-808-${(PO)}_{4}^{3-}$ | - | - | 2.16 |

## NMR, HPLC-MS and TGA data: (Fig. S1 - Fig. S10):

**Fig. S1.** ^1^H-NMR Spectra analysis for the glyphosate (GPh) degradation reaction, using nMOF-808 as heterogeneous catalyst. Potential product (formyl-glycine) structure analysis. The progress of GPh degradation was observed through integration, by the reduction of the area of its assigned signals (1H-NMR in D2O: δ = 3.25 ppm, doublet; and δ = 3.96 ppm, singlet), and the appearance of the signal assigned to formylglycine (1H-NMR in D2O: δ = 8.22 ppm, singlet; and δ = 2.08 ppm, singlet). These results allowed us to calculate a 95% and 77% removal of GPh for nMOF−808 and MOF-808, respectively. TON of 8.55 and 6.48 is calculated for nMOF-808 and MOF-808 per catalytic cycle (TON=n_product_/n_catalyst_)


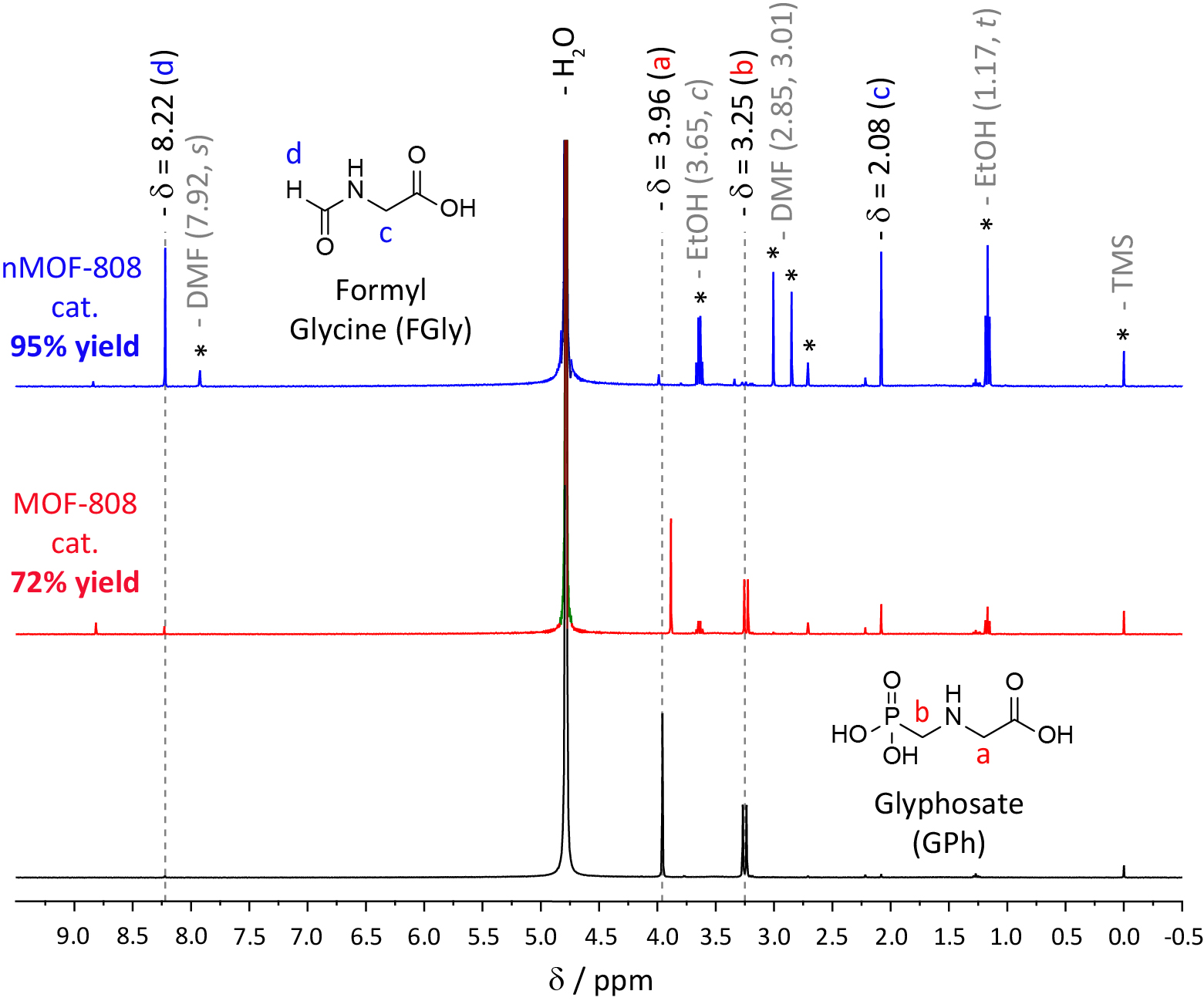


**Fig. S2.** High-performance liquid chromatography-mass spectrometry coupled to mass spectrometry (HPLC-Ms), calibration curve for glyphosate (GPh) degradation quantification. a) Table containing the different concentrations used in the calibration curve, and the areas corresponding to the GPh observation. b) Calibration curve graph, including the linear regression and its corresponding equation $\varepsilon_{1.15}$ = 47320 ppm-1. c) and d) LC chromatograms featuring retention times and peak areas for the observed GPh signal **after the degradation reaction is complete for MOF-808 and nMOF-808, respectively**.


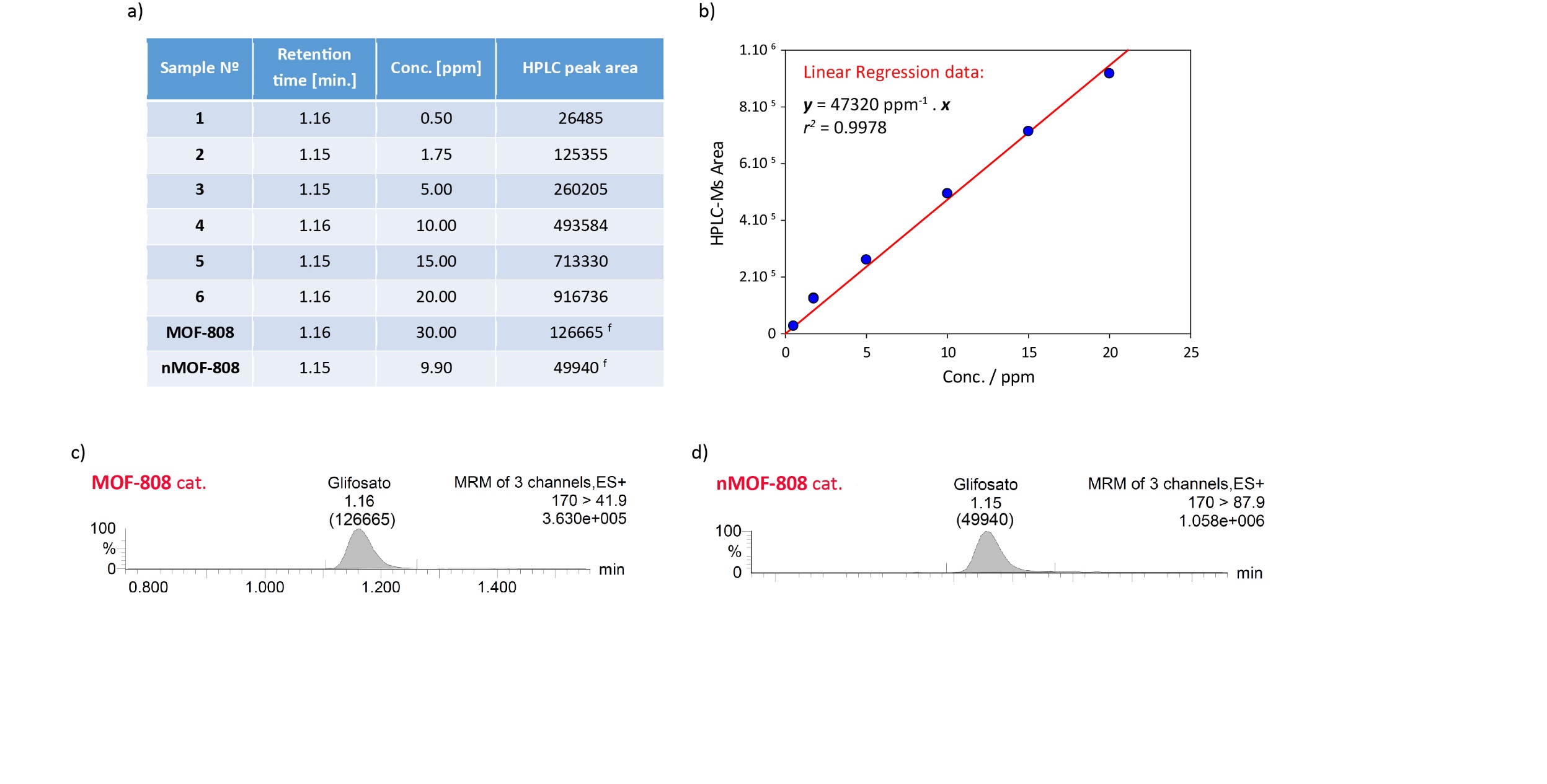


**Fig. S3.** ^13^C-NMR Spectrum analysis for the glyphosate (GPh) degradation product (formyl-glycine)

.


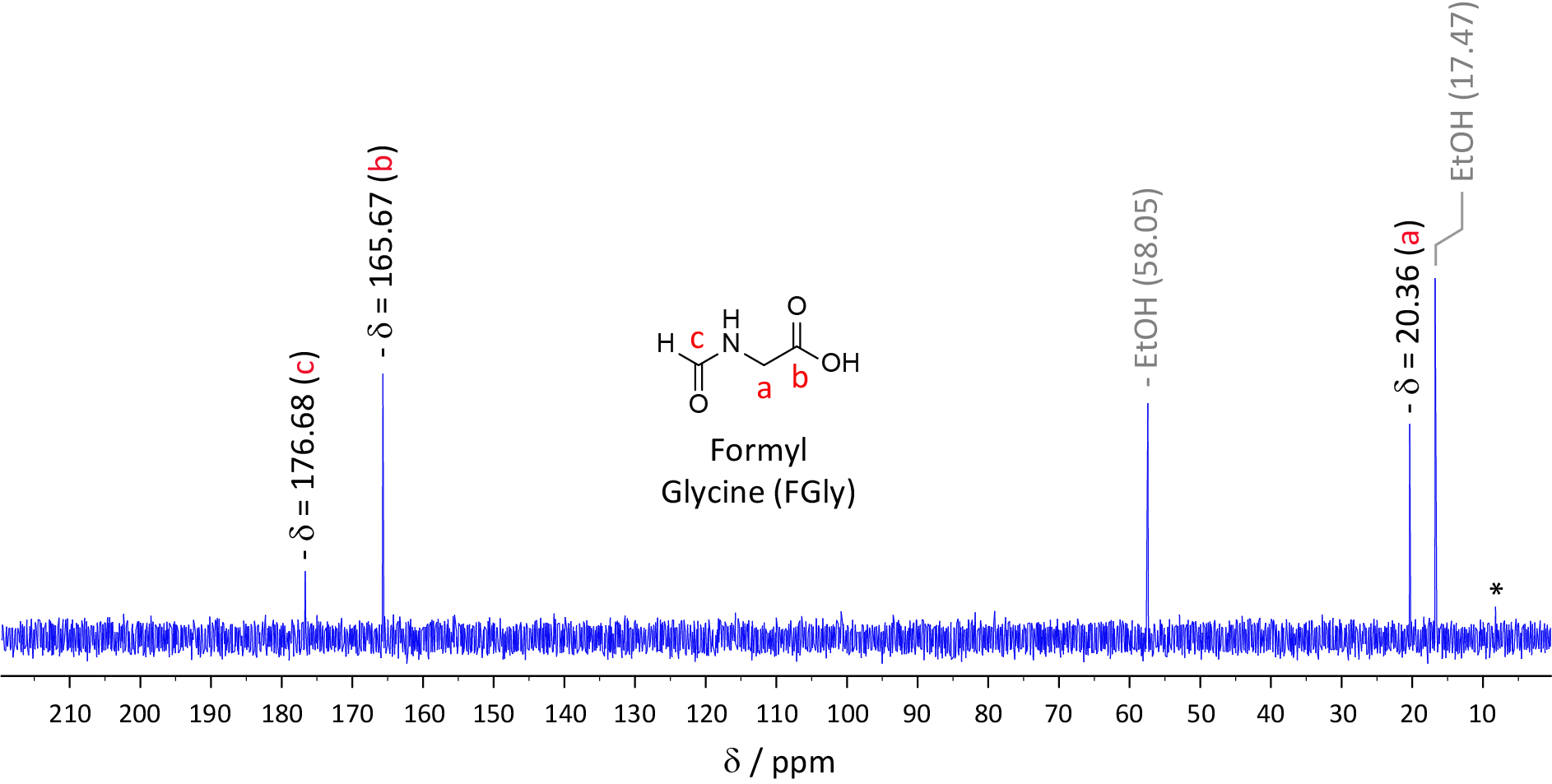


**Fig. S4.** Heteronuclear Multiple Bond Correlation Spectrum (HMBC) analysis for the glyphosate (GPh) degradation product (formyl-glycine).


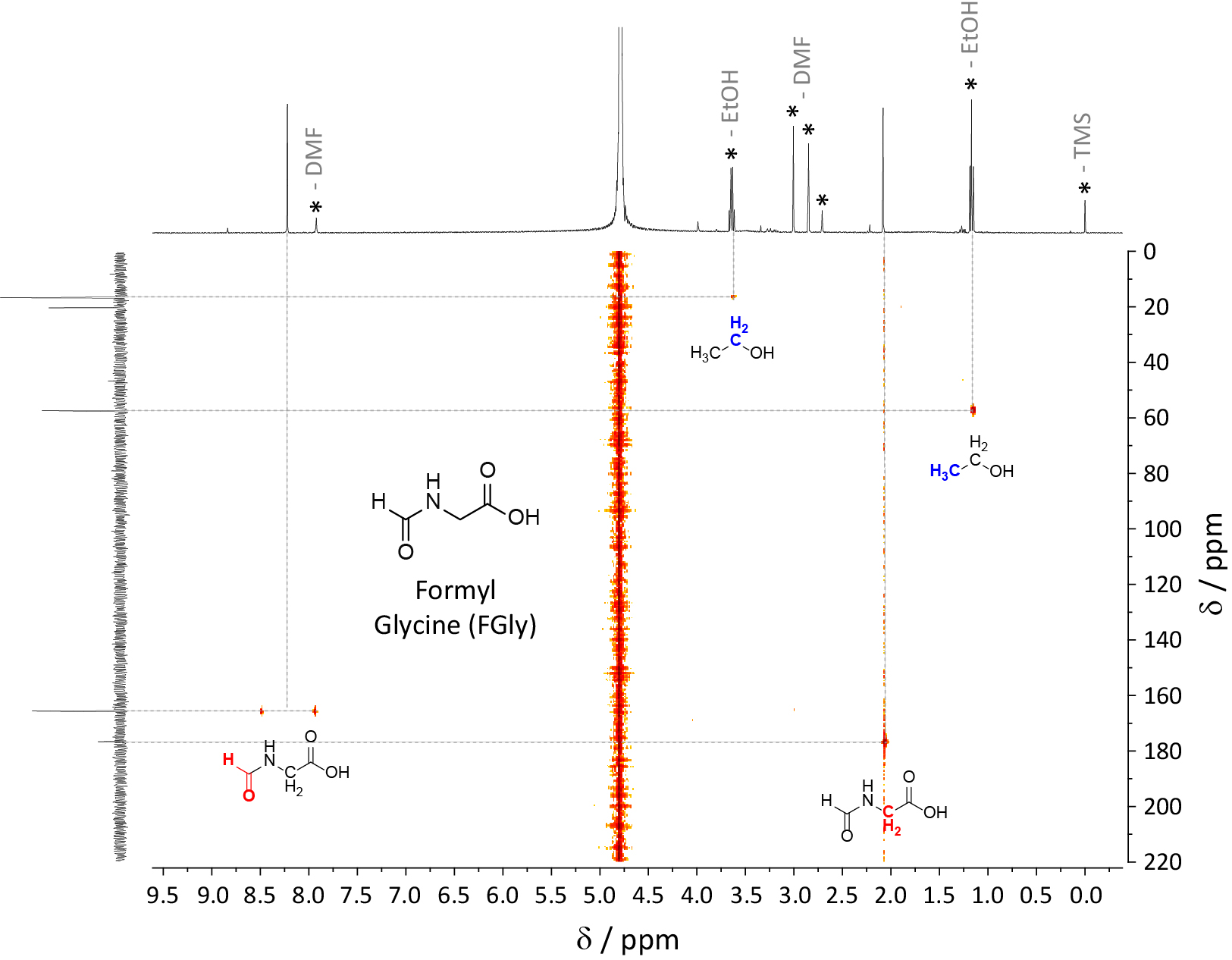


**Fig. S5.** ^1^H-NMR Spectrum analysis for the nMOF-808 reusability. We confirmed that the heterogeneous catalyst remains active in a second cycle for the GPh degradation reaction (ca. 91 % yield).


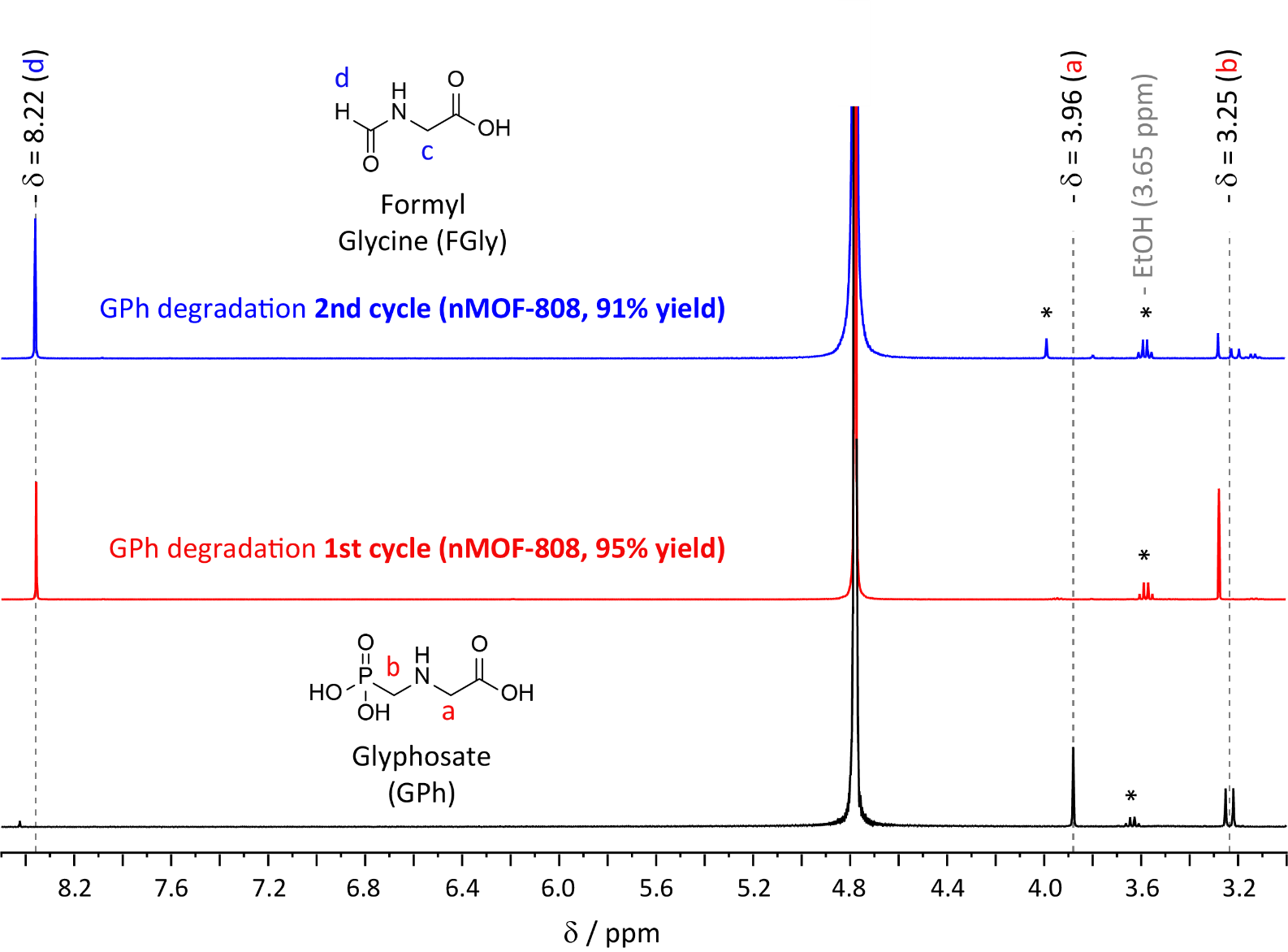


**Fig. S6.** ^31^P-NMR Spectrum analysis for the digested nMOF-808 after the first catalytic cycle. We can confirm that none of the found species attached to the MOF SUBs correspond to either phosphates or GPh P compounds.


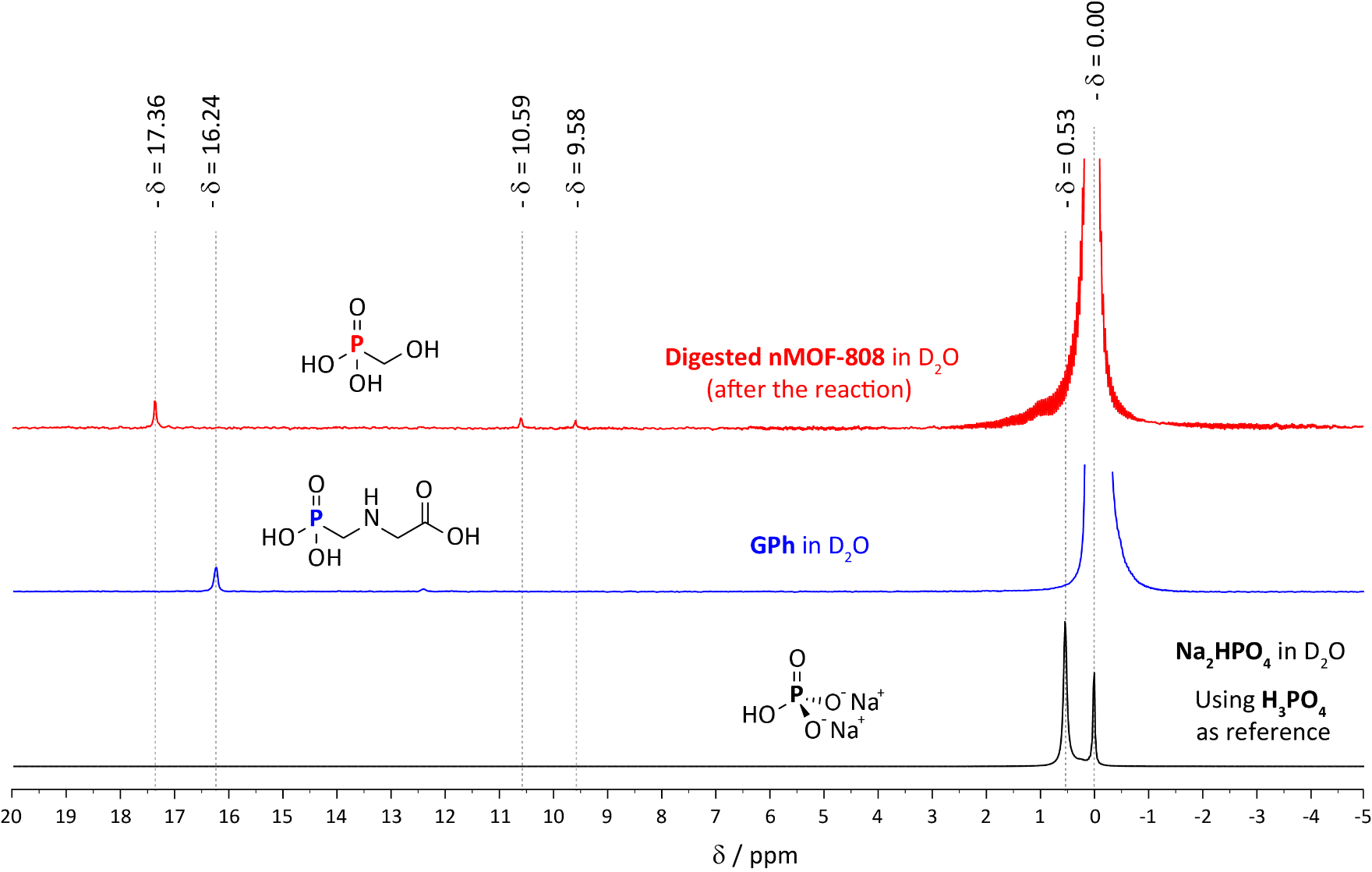


**Fig. S7.** Photocatalytic effects from ambient light are studied to confirm if there is any influence on the activity of GPh degradation by MOFs. a) Comparison of 1H-NMR spectra in D2O for: glyphosate used as substrate (GPh, black), D2O solution after 2 h of r.t. stirring in contact with nMOF-808 in the presence of ambient light (red spectrum), and D2O solution after 2 h of r.t. stirring in contact with nMOF-808 in the absence of ambient light (blue spectrum). The two reactions display GPh degradation >95%. b) Diffuse reflectance UV-Vis (DRUVS) spectra comparison for MOF-808 (red spectrum) and nMOF-808 catalysts (blue spectrum). The spectra display 2 absorption bands at 288 nm and 420 nm for both crystal sizes of MOF-808. The band presented at 288 nm is already reported^[28–32]^ showing limited light excitation effects restricted to ultraviolet wavelengths. Meanwhile, the band observed at 420 nm appears as a particular feature that can be assigned to an effect of deprotonation and coordination of trimesic acid^[33]^. These absorptions are not coupled with any electron transfer reactions, also it is observed that the catalytic performance is not affected in the absence of light. Hence catalytic properties are only associated to the structural and chemical properties of MOFs and its coordinated ligands around the Zr-SBUs at ambient conditions.


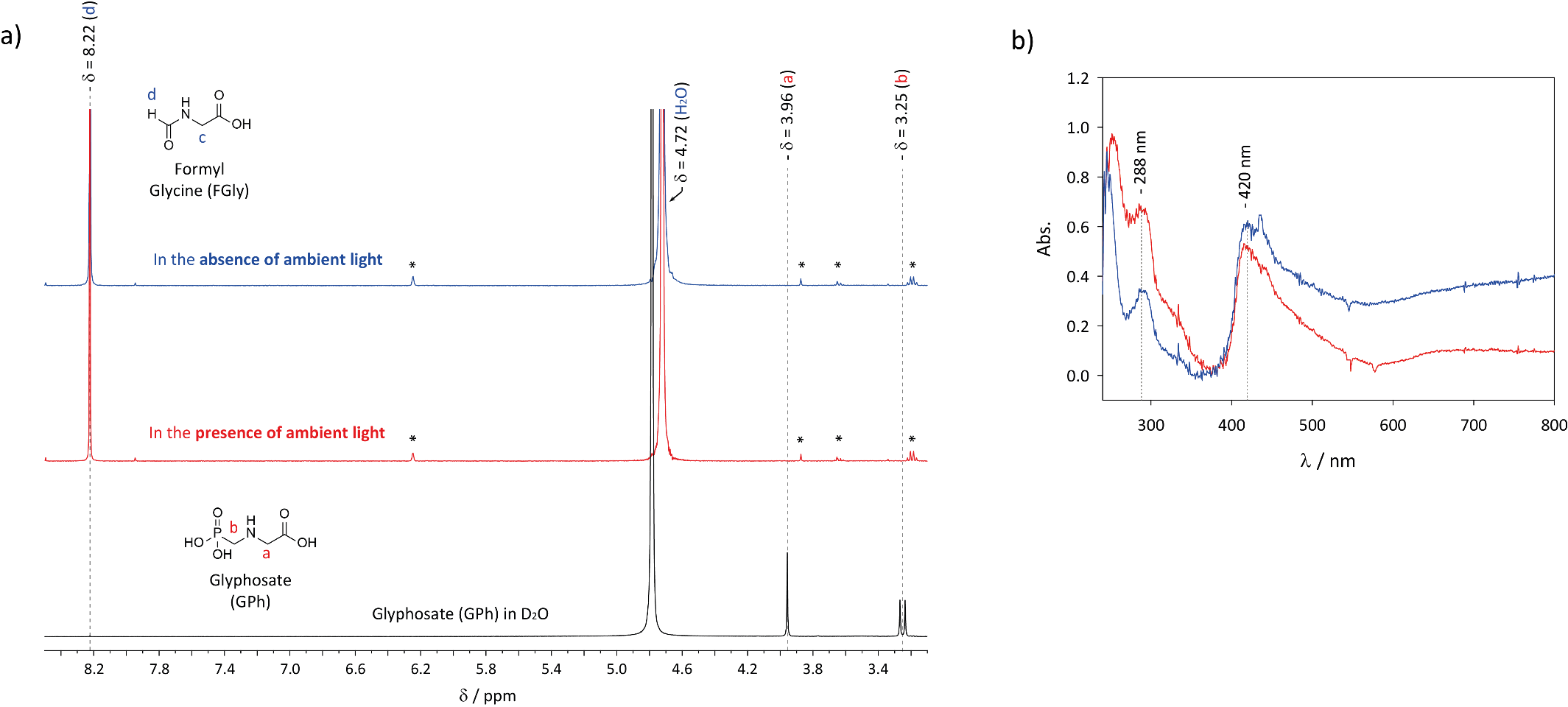


**Fig. S8.** ^1^H-NMR Spectra analysis for the glyphosate (GPh) degradation reaction, using Zr-oxo clusters as heterogeneous catalysts. 10% molar ratio of Zr oxo clusters to GPh (equivalent to 1.8 mg of Zr oxo cluster with 3.9 mg of GPh) in 1mL of D_2_O for 2 hours, at ambient temperature. The reaction is carried out also in 0.1 M of formic acid. The reaction is promoted by the presence of formates, but even in a concentrated HCOOH environment its performance is lower compared to the MOFs porous structures


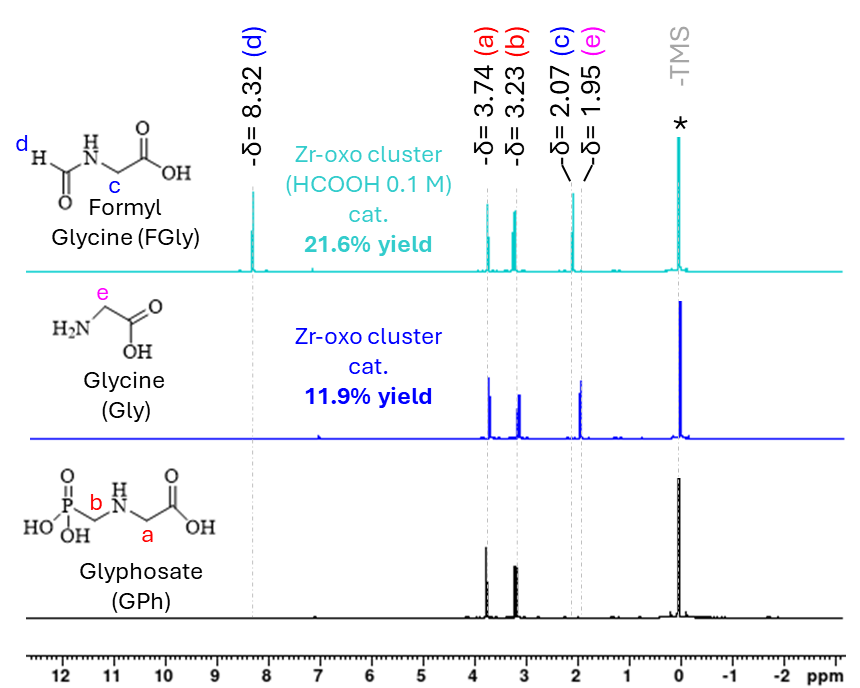


**Fig. S9.** Thermal gravimetric analysis (TGA) of pristine MOF-808 and nMOF-808. Concentration of defects calculated according to procedures reported^[34,35]^. The line at 600°C corresponds to wt.% of the Zr-SBU, which can be used to calculate the ideal weight percentage of the fully BTC coordinated MOFs using the FW. Zr_6_O_4_(OH)_4_(BTC)_2_. The values measured at 350 °C corresponds to wt.% of the experimental observed amount of BTC linkers of each MOF and defects concentration can be calculated using formula (1). A higher number of defects is observed for nMOF-808

**
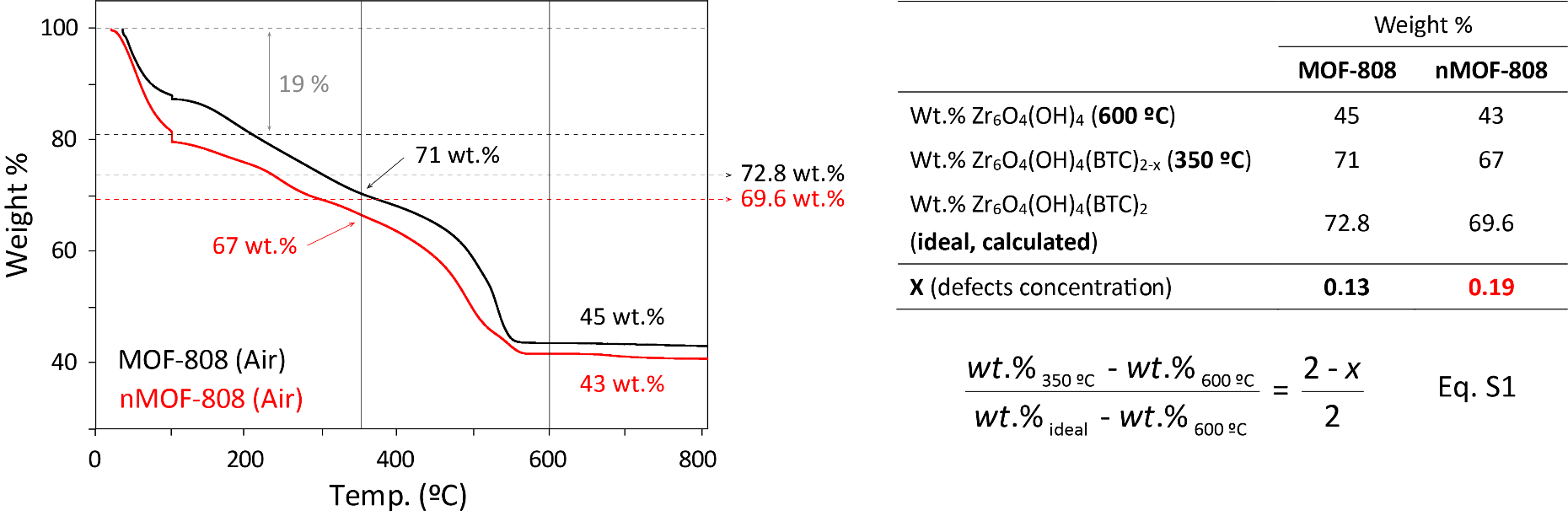
**

**Fig. S10.** Mass spectrum, for the obtained product from GPh degradation, formyl-glycine (its derivative produced under the ionization conditions).

**
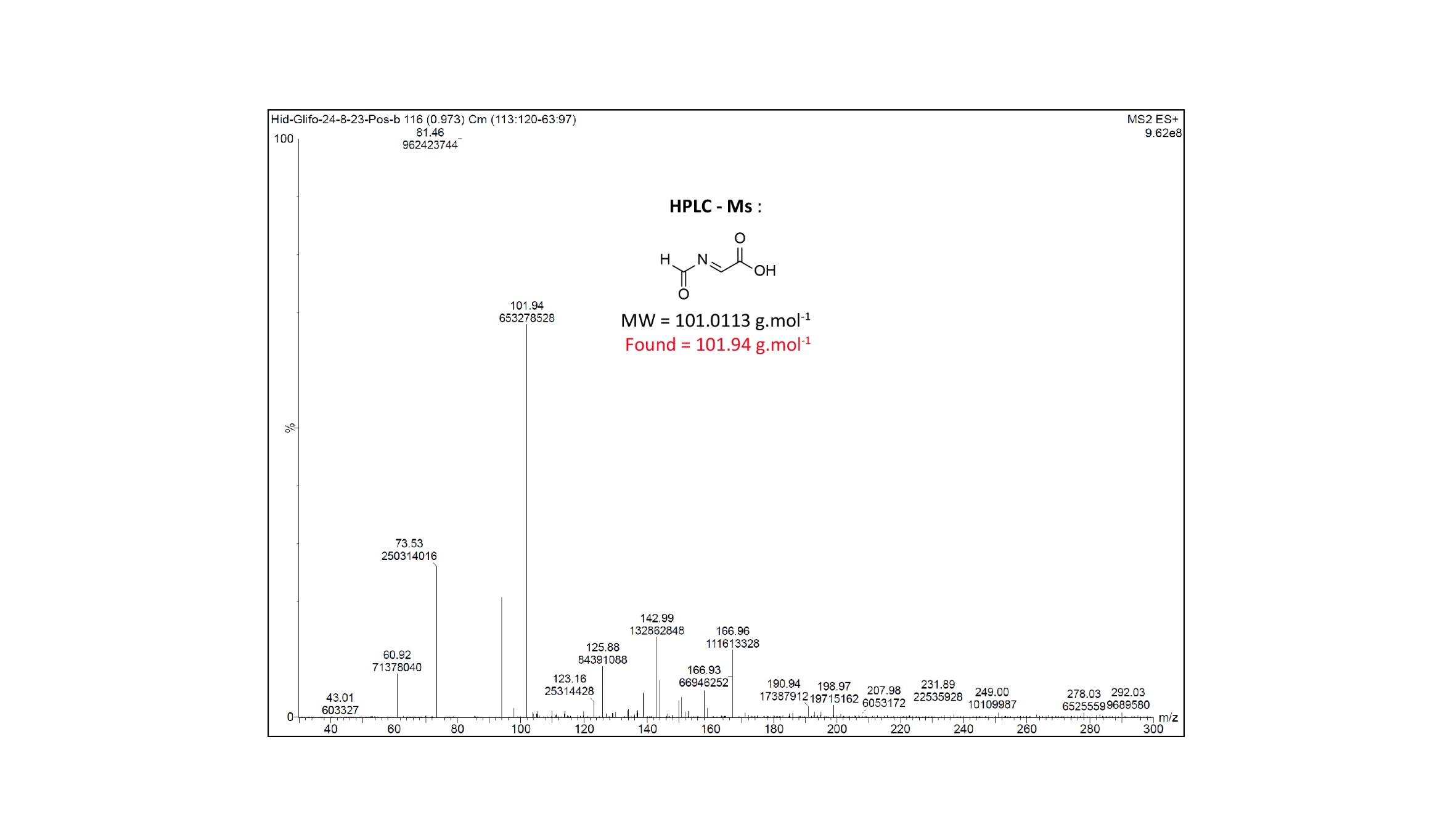
**

## Synchrotron data (XAS and total scattering): (Fig. S11 - S18)

**Fig. S11.** Reaction progress of degradation test of MOF-808 with p-Nitrophneylphosphate (pNPP) towards p-nitrophenol (pNP) and phosphoric acid. a) UV-Vis spectra showing the degradation of pNPP (absorption band 311 nm) to pNP (absorption band 403 nm). b) P K-edge spectra of standard materials glyphosate (GPh), pNPP, K2HPO4 and recovered MOF-808 after reaction with pNPP named MOF-808-PO43-


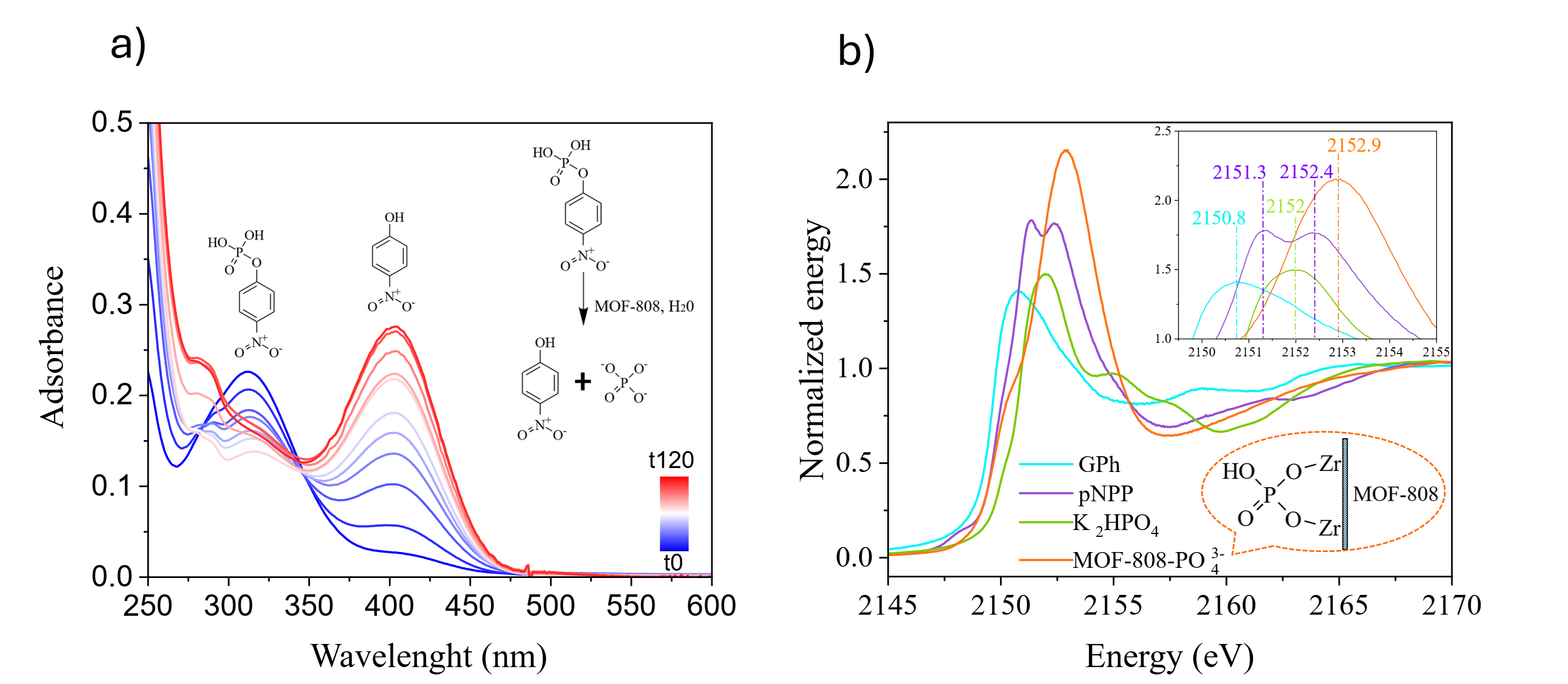


**Fig. S12.** C K-edge NEXAFS data analysis for a) the pristine (MOF-808 and nMOF-808) and the recovered after reaction MOFs (MOF-808-GPh and nMOF−808-GPh) and b) Reference samples of the Zr-Oxo (Zr oxocluster used during the nMOF-808 synthesis), trimesic acid (BTC) and glyphosate (GPh).


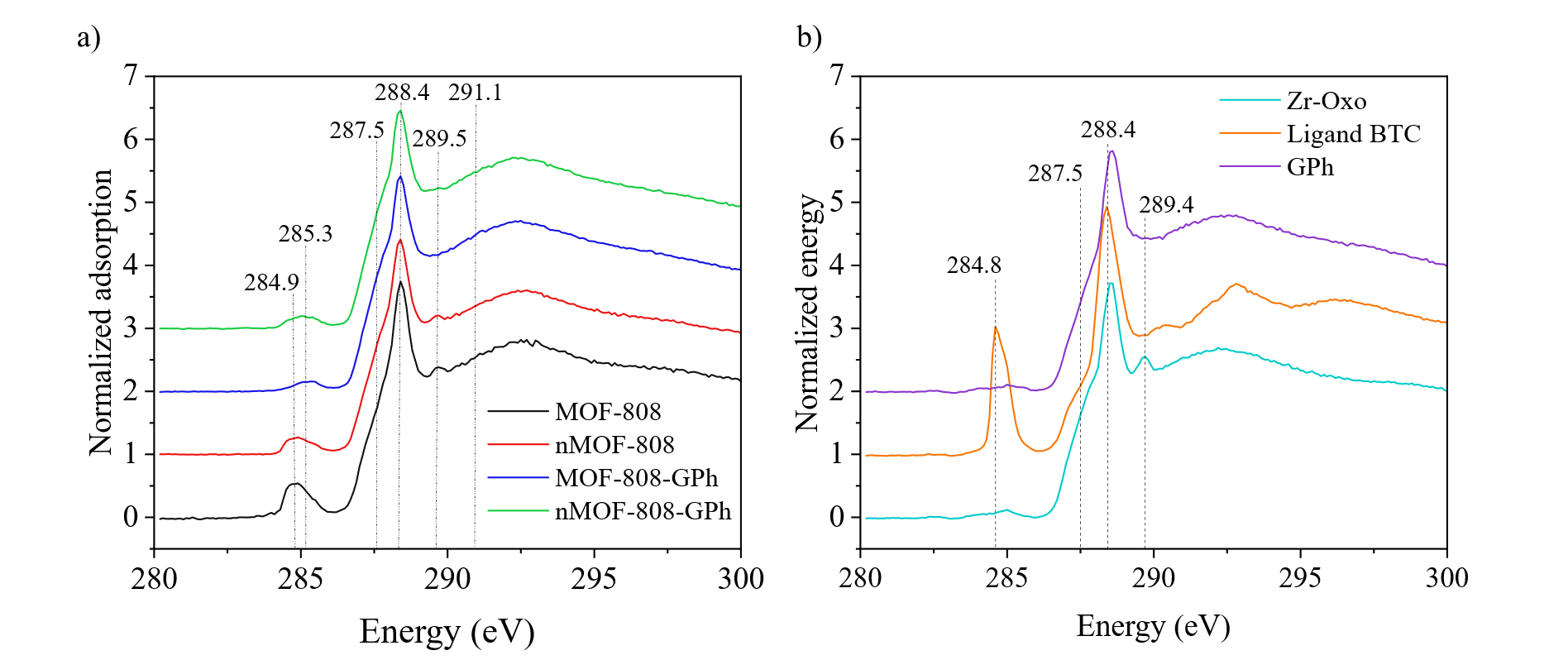


**Fig. S13.** C K-edge NEXAFS deconvolution analysis results of pristine (MOF-808 and nMOF-808) and the recovered post-reaction MOFs (MOF-808-GPh and nMOF-808-GPh). a) Deconvolution of C K-edge spectra into Gaussian curves, b) Functional groups associated with their corresponding energy peak transition.


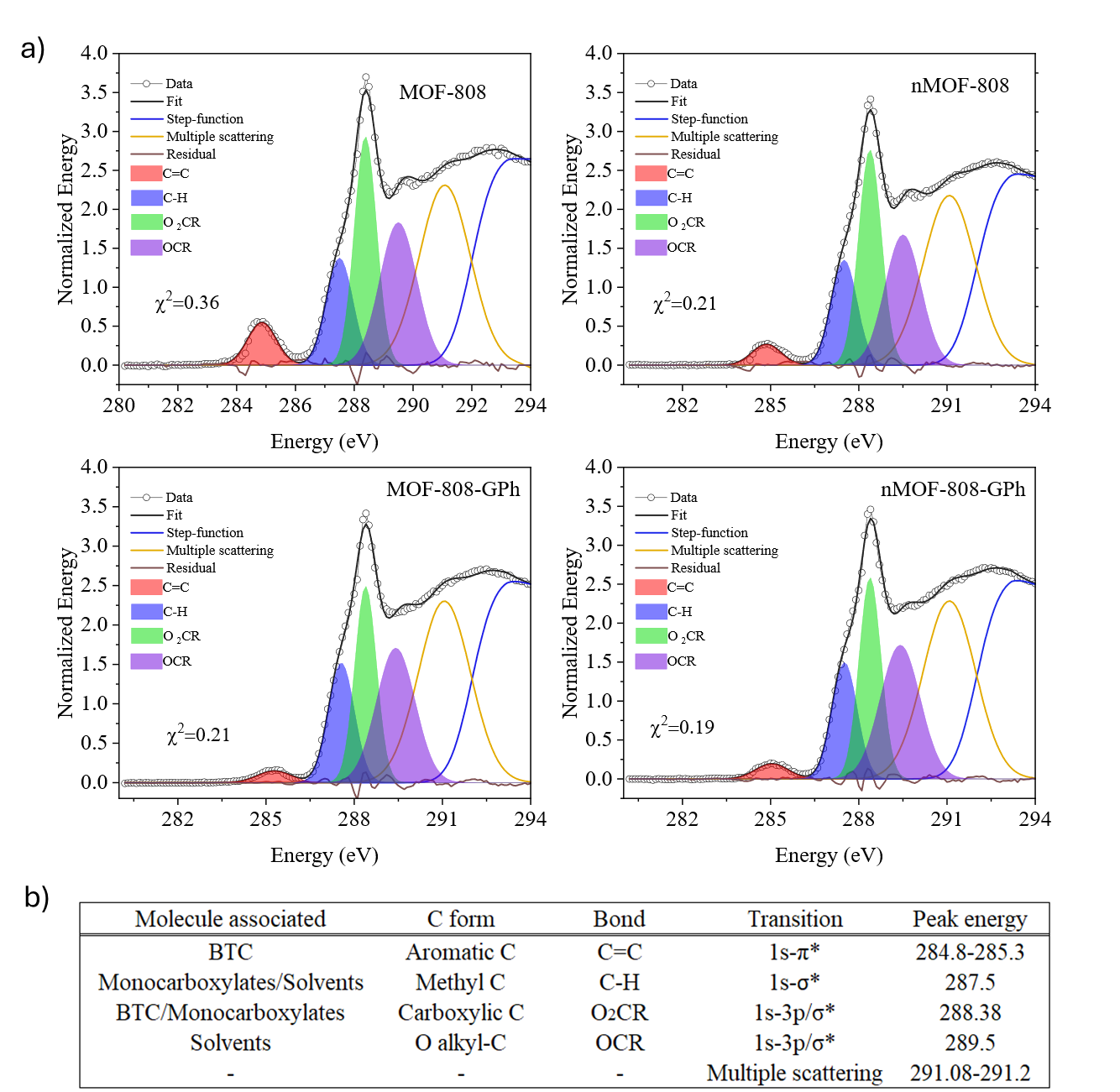


**Fig. S14.** O K-edge NEXAFS data analysis for a) the pristine (MOF-808 and nMOF-808) and the recovered post-reaction MOFs (MOF-808-GPh and nMOF−808-GPh) and b) Reference samples of the Zr-Oxo (Zr oxocluster used during the nMOF-808 synthesis), trimesic acid (BTC) and glyphosate (GPh).


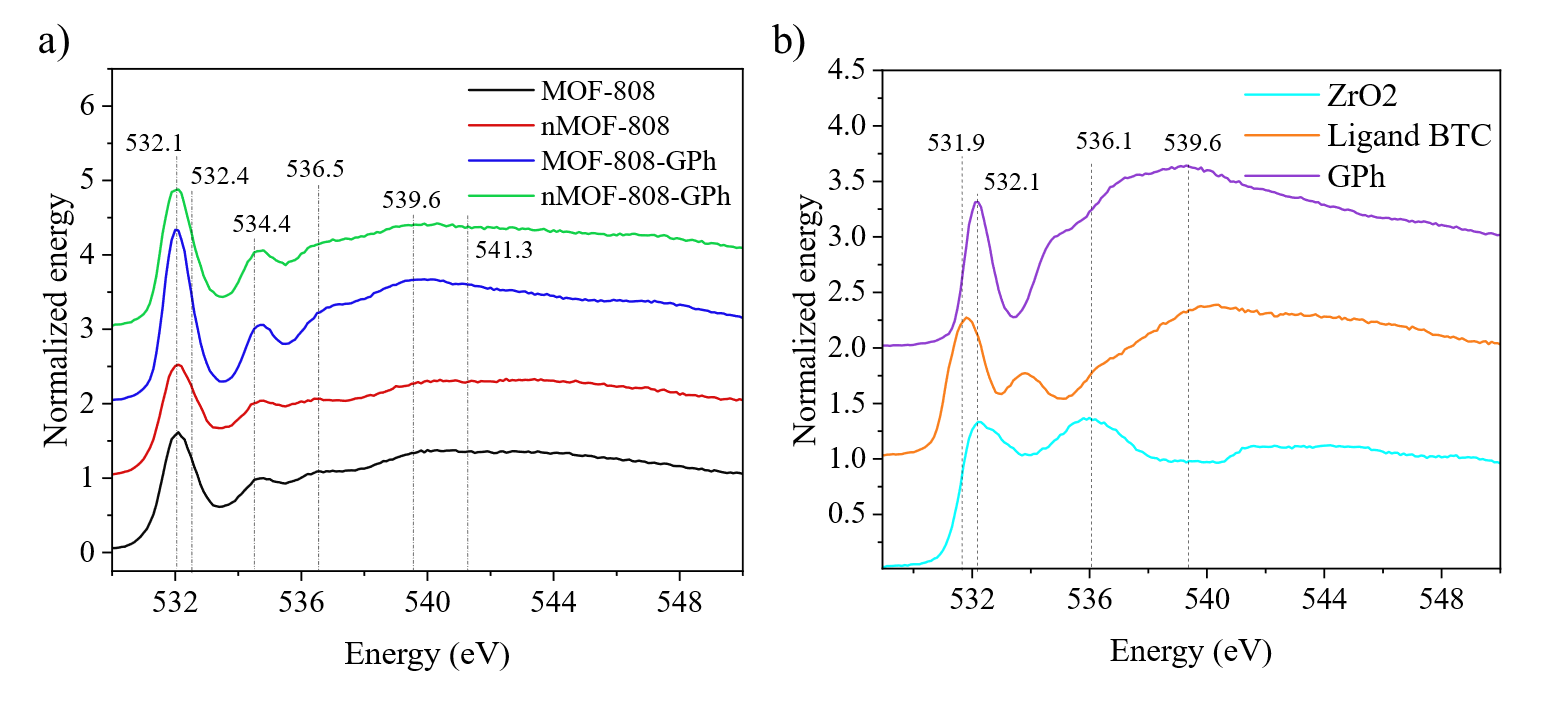


**Fig. S15.** O K-edge NEXAFS deconvolution analysis of pristine (MOF-808 and nMOF-808) and the recovered post-reaction MOFs (MOF-808-GPh and nMOF-808-GPh). a) Deconvolution of C K-edge spectra into gaussian curves, b) Functional groups associated with their corresponding energy peak transition


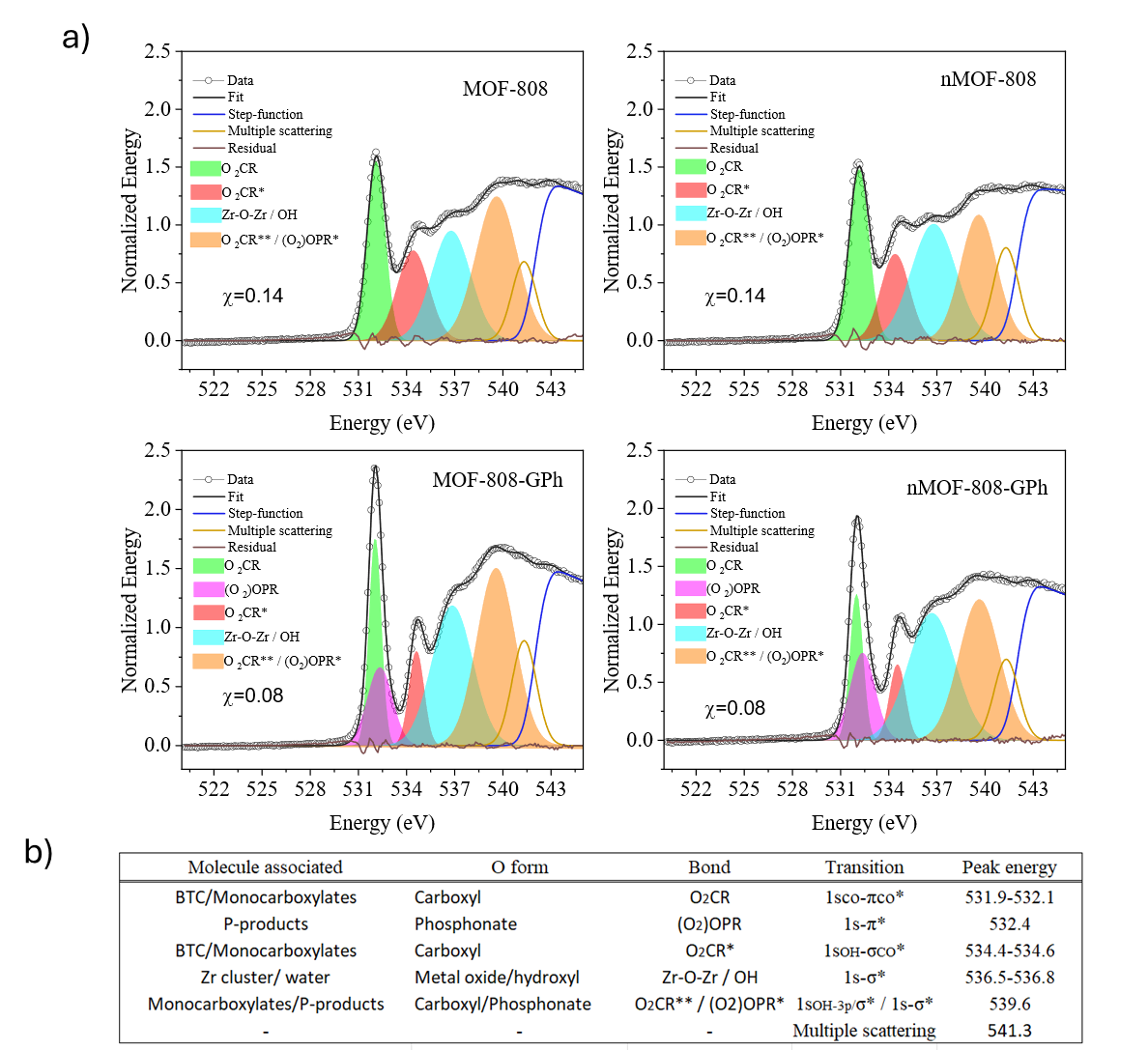


**Fig. S16.** PDF analysis of pre and post reaction MOFs materials qmax=27. a) Pristine MOF-808 and nMOF-808. b) Gaussian fit of pristine MOFs on the Zr-Zr and Zr-O distances. c) PDFs of all samples plus normalized differential PDFs obtained by subtracting pair contributions of the pristine MOFs from the corresponding post-reaction material.


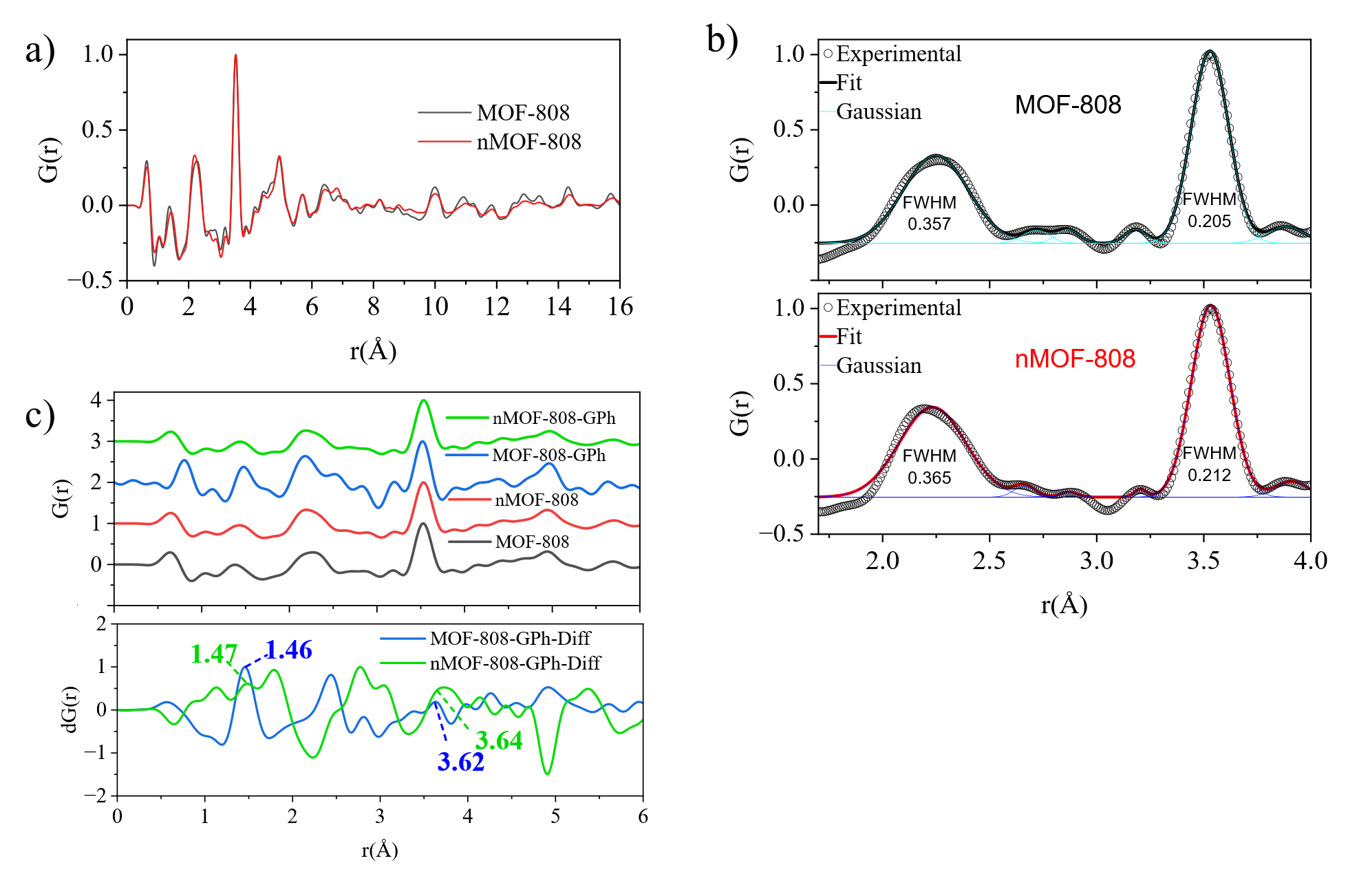


**Fig. S17.** Zr K-edge FT-EXAFS fitting of pristine MOF-808 and nMOF-808 (K weight 2; Δk = 3.0-11.3 Å^-1^; ΔR = 1.0-4.0 Å)


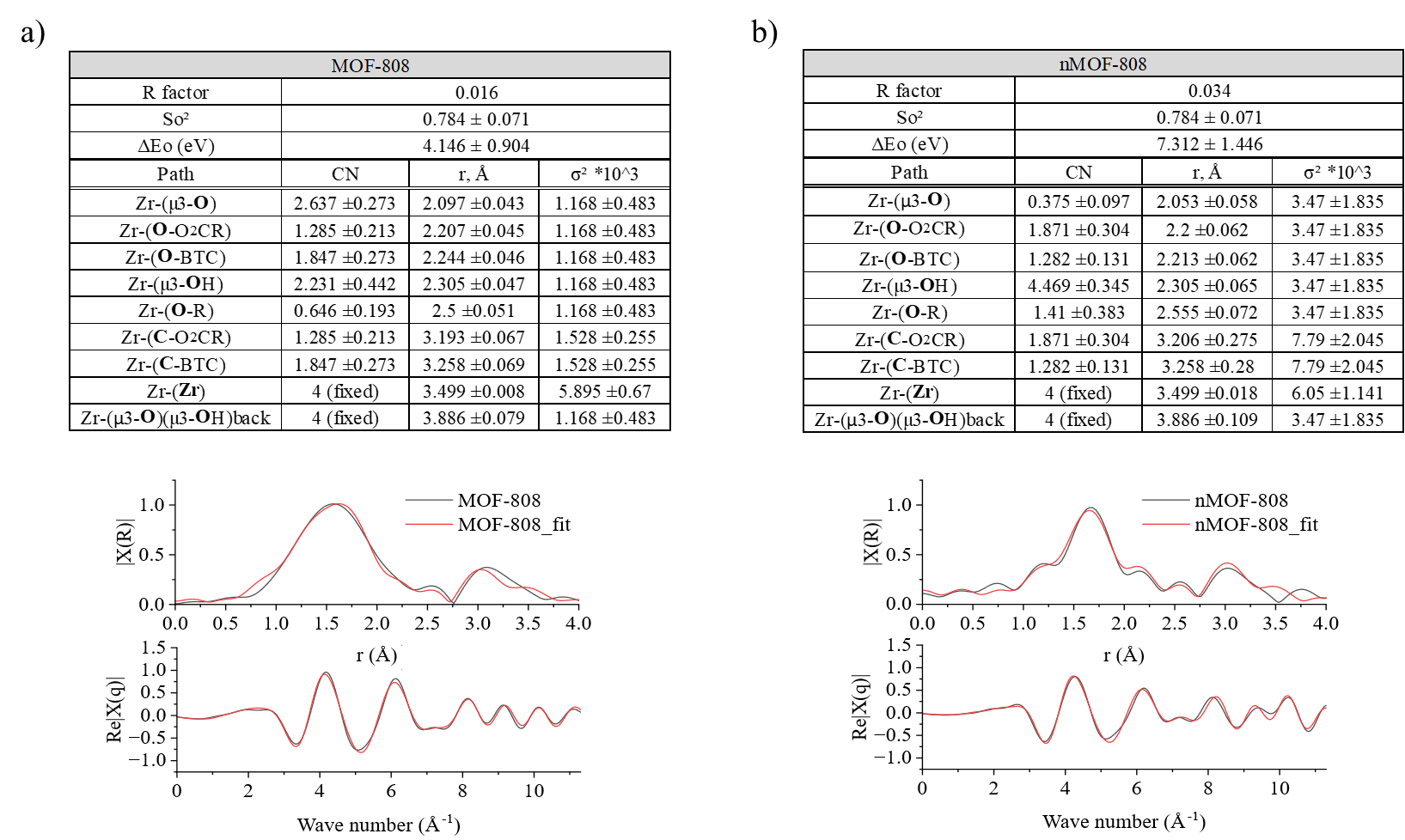


**Fig. S18.** Zr K-edge FT-EXAFS fitting of post-reaction MOF-808-GPh and nMOF-808-GPh (K weight 2; Δk = 3.0-11.3 Å^-1^; ΔR = 1.0-4.0 Å)


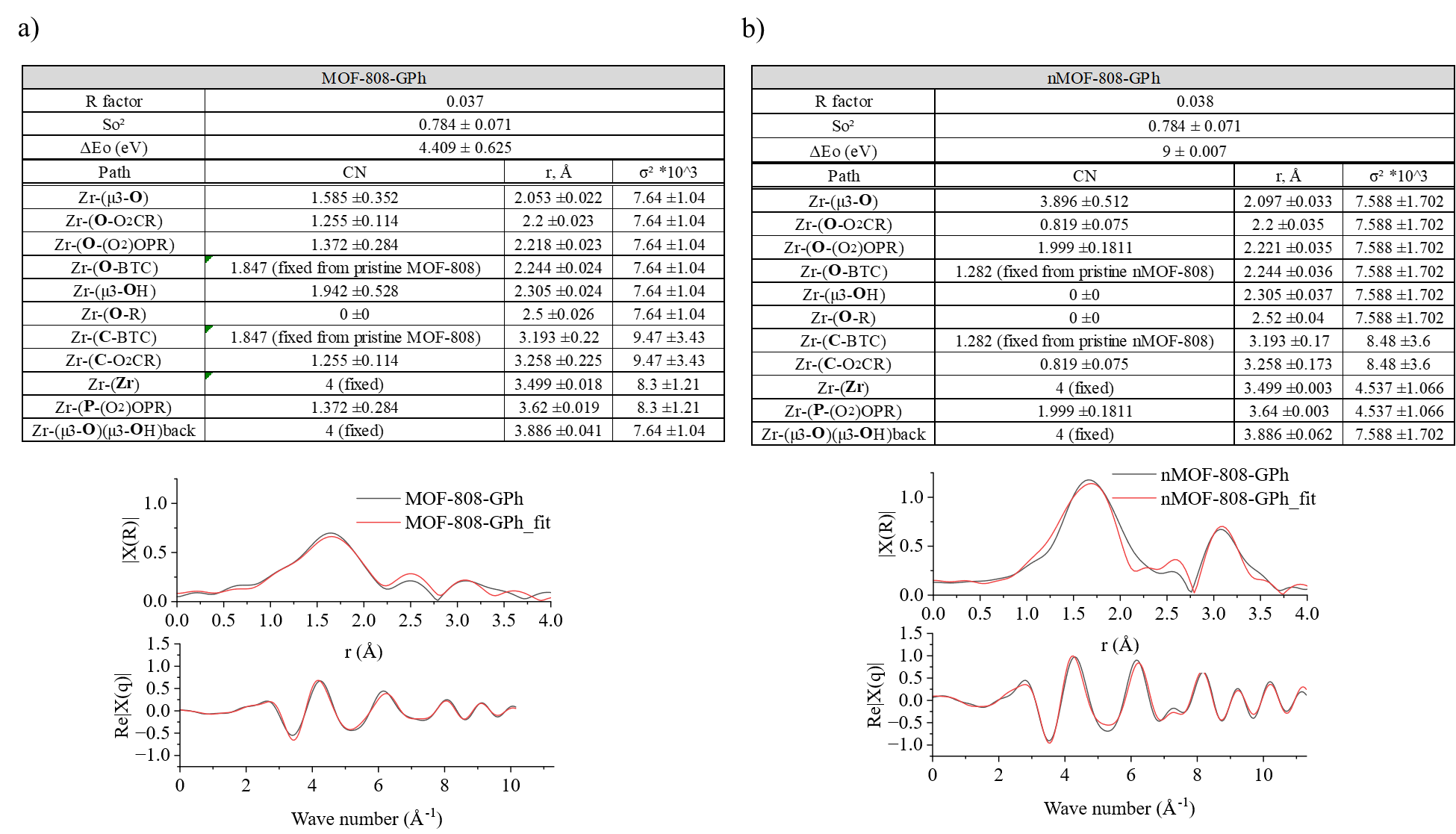


## Experiments with formic acid (Fig. S19 – S21)

**Fig. S19**. ^1^H-NMR Spectra analysis for the glyphosate (GPh) degradation reaction (1 hour), using nMOF-808 as heterogeneous catalyst with and without HCOOH in solution (HCOOH concentration, 0.01 M _ pH 2.9 and 1e-5 M _ pH 4.4). Peak of the carboxyl group of FGly (δ=8.32 ppm) in the experiment of nMOF-808 at HCOOH 0.01M is overlapped by formic acid.


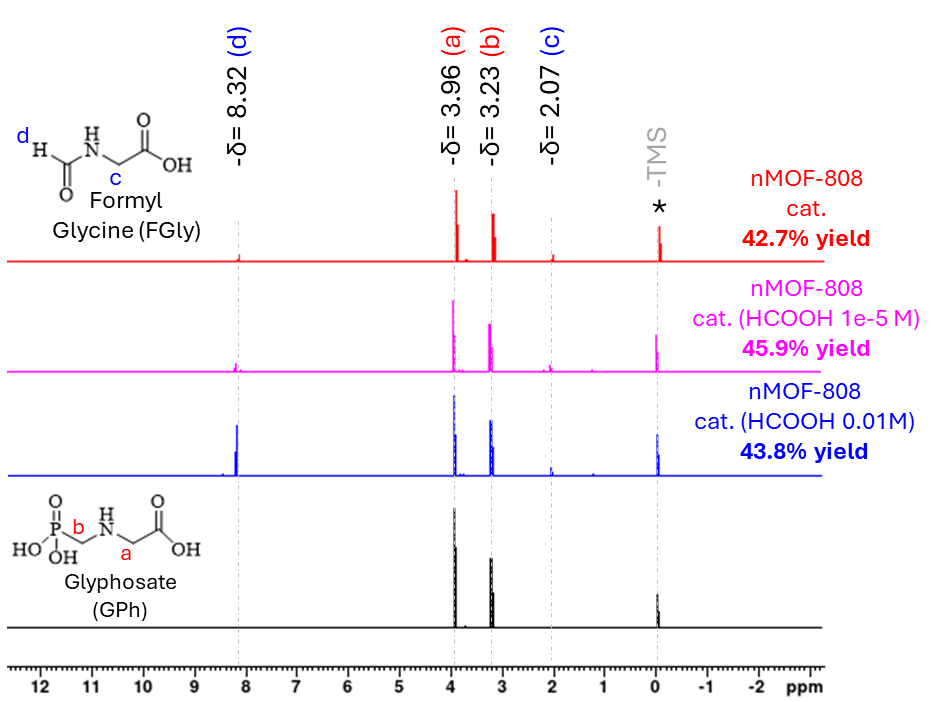


**Fig S20.** a) ^1^H-NMR Spectra analysis for digested nMOF-808 and nMOF-808-GPh (pristine and after reaction). 10% molar ratio of MOF to GPh (equivalent to 2 mg of nMOF-808 with 2.2 mg of GPh) in 1mL of D2O for 2 hours, at ambient temperature. b) calibration curve of integrated peak areas of ^1^H-NMR at δ=8.46ppm of HCOOH and calculated concentration of digested MOFs from spectra of a). Significant amounts of formates are observed in both MOFs materials to continue for consecutive reactions. c) an estimation of 8.38 molar ratio of Hydroxymethyl phosphonate/nMOF-808 is calculated using the TMS concentration (TMS 0.01M, C1/A1=C2/A2) confirming that all phosphonate product remains trapped in the MOF (TON 8.55, Fig S1).


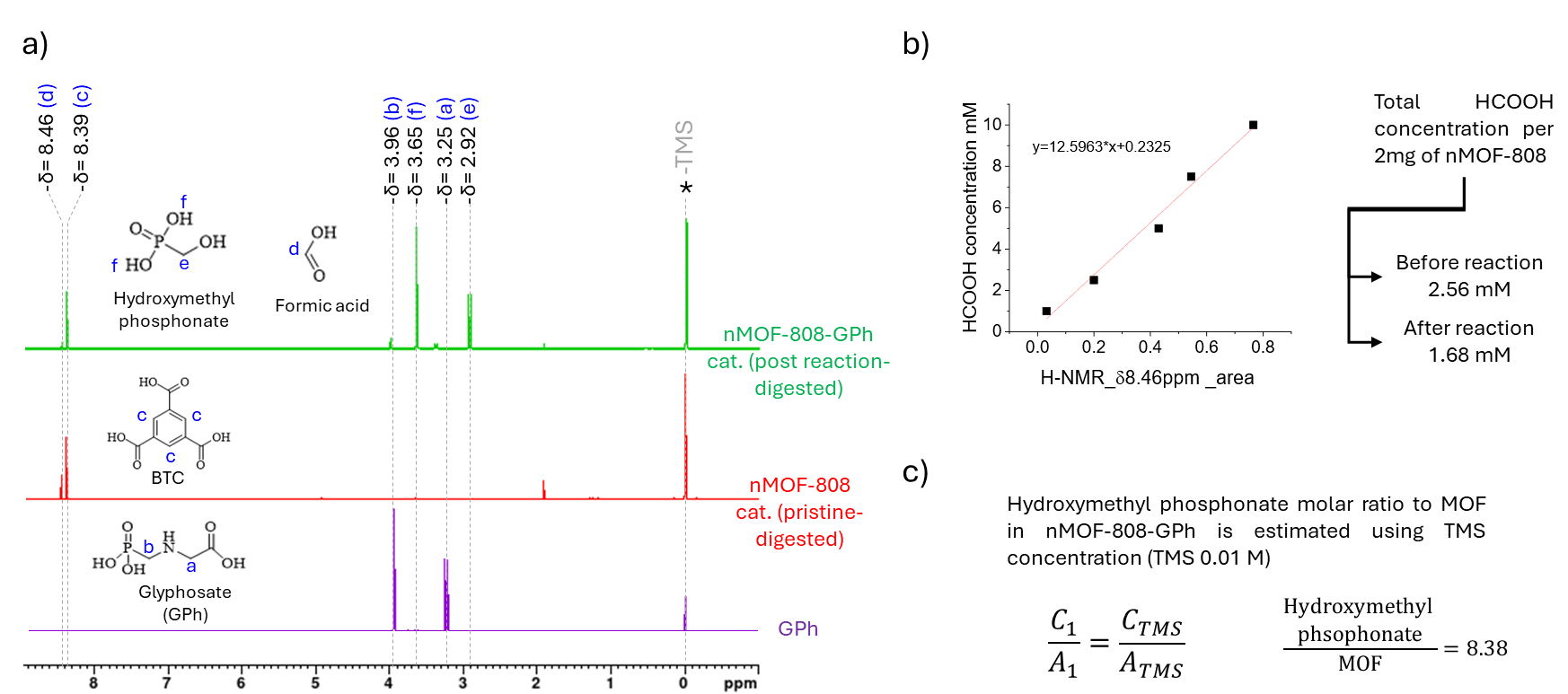


**Fig S21**. a) ^1^H-NMR Spectra analysis for digested MOF-808 and MOF-808-GPh (pristine and after reaction). 10% molar ratio of MOF to GPh (equivalent to 2 mg of MOF-808 with 2.2 mg of GPh) in 1mL of D2O for 2 hours, at ambient temperature. b) calibration curve of integrated peak areas of ^1^H-NMR at δ=8.46ppm of HCOOH and calculated concentration of digested MOFs from spectra of a). Significant amounts of formates are observed in both MOFs materials to continue for consecutive reactions. c) an estimation of 6.81 molar ratio of Hydroxymethyl phosphonate/nMOF-808 is calculated using the TMS concentration (TMS 0.01M, C1/A1=C2/A2) confirming that all phosphonate product remains trapped in the MOF (TON 6.48, Fig S1).


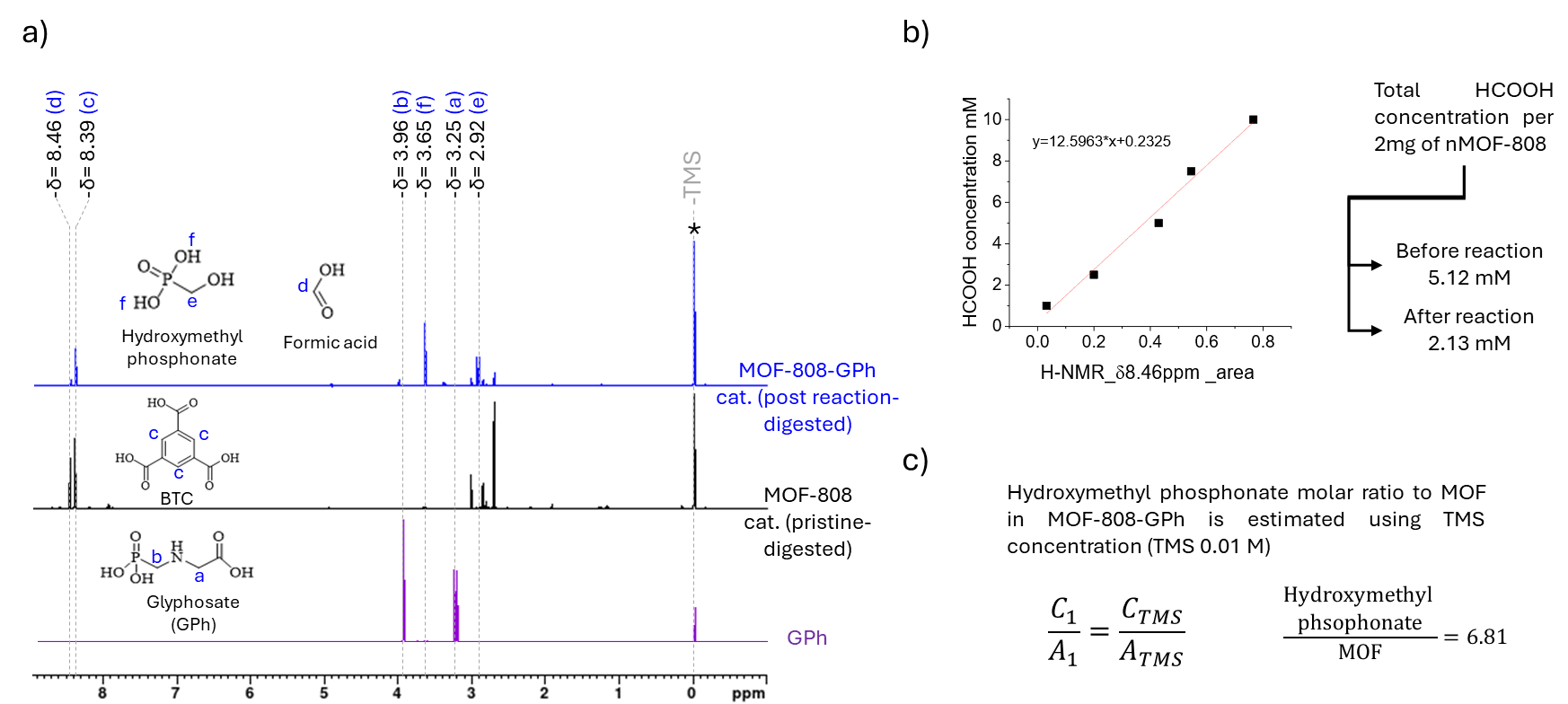


# References

[1] H. Furukawa, F. Gándara, Y. B. Zhang, J. Jiang, W. L. Queen, M. R. Hudson, O. M. Yaghi, *J Am Chem Soc* **2014**, *136*, 4369–4381.

[2] S. Dai, C. Simms, I. Dovgaliuk, G. Patriarche, A. Tissot, T. N. Parac-Vogt, C. Serre, *Chemistry of Materials* **2021**, *33*, 7057–7066.

[3] E. Gann, C. R. McNeill, A. Tadich, B. C. C. Cowie, L. Thomsen, *J Synchrotron Radiat* **2016**, *23*, 374–380.

[4] B. Ravel, M. Newville, *J Synchrotron Radiat* **2005**, *12*, 537–541.

[5] P. Juhás, T. Davis, C. L. Farrow, S. J. L. Billinge, *J Appl Crystallogr* **2013**, *46*, 560–566.

[6] J. Henych, M. Šťastný, Z. Němečková, K. Mazanec, J. Tolasz, M. Kormunda, J. Ederer, P. Janoš, *Chemical Engineering Journal* **2021**, *414*, 128822.

[7] S. Li, Z. Zhou, Z. Tie, B. Wang, M. Ye, L. Du, R. Cui, W. Liu, C. Wan, Q. Liu, S. Zhao, Q. Wang, Y. Zhang, S. Zhang, H. Zhang, Y. Du, H. Wei, *Nat Commun* **2022**, *13*, DOI 10.1038/s41467-022-28344-2.

[8] T. Li, W. Zhong, C. Jing, X. Li, T. Zhang, C. Jiang, W. Chen, *Environ Sci Technol* **2020**, *54*, 8658–8667.

[9] P. Paudel, A. Negusse, D. P. Jaisi, *Soil Science Society of America Journal* **2015**, *79*, 815–825.

[10] Y. Chen, Y. Huang, H. Tian, L. Ye, R. Li, C. Chen, Z. Dai, D. Huang, *Journal of Environmental Sciences* **2023**, *127*, 60–68.

[11] L. Jin, Y. Chen, H. Tian, X. Liu, Y. Huang, R. Li, C. Chen, Z. Dai, D. Huang, *Sep Purif Technol* **2023**, *320*, 124164.

[12] D. Feng, A. Soric, O. Boutin, *Science of the Total Environment* **2020**, *742*, DOI 10.1016/j.scitotenv.2020.140559.

[13] A. Manassero, C. Passalia, A. C. Negro, A. E. Cassano, C. S. Zalazar, *Water Res* **2010**, *44*, 3875–3882.

[14] I. Persson, W. Klysubun, D. Lundberg, *J Mol Struct* **2019**, *1179*, 608–611.

[15] A. J. Lopes Catão, A. López-Castillo, *Environ Sci Process Impacts* **2018**, *20*, 1148–1157.

[16] C. Ehlert, W. E. S. Unger, P. Saalfrank, *Physical Chemistry Chemical Physics* **2014**, *16*, 14083–14095.

[17] K. Heymann, J. Lehmann Johannes, D. Solomon, M. W. I. Schmidt, T. Regier, *Org Geochem* **2011**, *42*, 1055–1064.

[18] K. Shibata, K. Kikumasa, S. Kiyohara, T. Mizoguchi, *Sci Data* **2022**, *9*, DOI 10.1038/s41597-022-01303-8.

[19] A. D. Winter, E. Larios, F. M. Alamgir, C. Jaye, D. Fischer, E. M. Campo, *Langmuir* **2013**, *29*, 15822–15830.

[20] A. Ganguly, S. Sharma, P. Papakonstantinou, J. Hamilton, *The Journal of Physical Chemistry C* **2011**, *115*, 17009–17019.

[21] A. Nandy, A. C. Forse, V. J. Witherspoon, J. A. Reimer, *The Journal of Physical Chemistry C* **2018**, *122*, 8295–8305.

[22] F. Frati, M. O. J. Y. Hunault, F. M. F. De Groot, *Chem Rev* **2020**, *120*, 4056–4110.

[23] K. Kim, P. Zhu, N. Li, X. Ma, Y. Chen, *Carbon N Y* **2011**, *49*, 1745–1751.

[24] B. M. Messer, C. D. Cappa, J. D. Smith, K. R. Wilson, M. K. Gilles, R. C. Cohen, R. J. Saykally, *Journal of Physical Chemistry B* **2005**, *109*, 5375–5382.

[25] J. Rajendran, S. Gialanella, P. B. Aswath, *Materials Science and Engineering C* **2013**, *33*, 3968–3979.

[26] K. Tabayashi, K. Yamamoto, O. Takahashi, Y. Tamenori, J. R. Harries, T. Gejo, M. Iseda, T. Tamura, K. Honma, I. H. Suzuki, S. I. Nagaoka, T. Ibuki, *Journal of Chemical Physics* **2006**, *125*, DOI 10.1063/1.2387949.

[27] D. Troya, *The Journal of Physical Chemistry C* **2016**, *120*, 29312–29323.

[28] C. Castillo-Blas, M. J. García, A. M. Chester, M. Mazaj, S. Guan, G. P. Robertson, A. Kono, J. M. A. Steele, L. León-Alcaide, B. Poletto-Rodrigues, P. A. Chater, S. Cabrera, A. Krajnc, L. Wondraczek, D. A. Keen, J. Alemán, T. D. Bennett, *ACS Appl Mater Interfaces* **2025**, DOI 10.1021/acsami.4c18444.

[29] Y. Dong, X. Wang, H. Sun, H. Zhang, X. Zhao, L. Wang, *Chemical Engineering Journal* **2023**, *460*, DOI 10.1016/j.cej.2023.141842.

[30] M. Rahmani, A. Abbasi, M. S. Hosseini, *Appl Surf Sci* **2024**, *648*, DOI 10.1016/j.apsusc.2023.159014.

[31] J. Xu, J. Liu, Z. Li, X. Wang, Z. Wang, *J Mater Sci* **2019**, *54*, 12911–12924.

[32] F. Wang, R. Xue, Y. Ma, Y. Ge, Z. Wang, X. Qiao, P. Zhou, *RSC Adv* **2021**, *11*, 32955–32964.

[33] T. Alammar, I. Z. Hlova, S. Gupta, V. Balema, V. K. Pecharsky, A. V. Mudring, *Dalton Transactions* **2018**, *47*, 7594–7601.

[34] G. Kaur, S. Øien-ØDegaard, A. Lazzarini, S. M. Chavan, S. Bordiga, K. P. Lillerud, U. Olsbye, *Cryst Growth Des* **2019**, *19*, 4246–4251.

[35] J. Yin, Z. Kang, Y. Fu, W. Cao, Y. Wang, H. Guan, Y. Yin, B. Chen, X. Yi, W. Chen, W. Shao, Y. Zhu, A. Zheng, Q. Wang, X. Kong, *Nat Commun* **2022**, *13*, 5112.
